# Phytoplankton competition and resilience under fluctuating temperature

**DOI:** 10.1002/ece3.9851

**Published:** 2023-03-19

**Authors:** Philipp Siegel, Kirralee G. Baker, Etienne Low‐Décarie, Richard J. Geider

**Affiliations:** ^1^ School of Life Sciences University of Essex Colchester Campus Colchester UK; ^2^ Present address: Institute for Marine and Antarctic Studies University of Tasmania Battery Point Tasmania Australia; ^3^ Present address: Biological Informatics Center of Expertise, Agriculture and Agrifoods Canada, Government of Canada Montreal Quebec Canada

**Keywords:** climate change, diatom, disturbance, heatwaves, nitrogen limitation, *Phaeodactylum tricornutum*, *Thalassiosira pseudonana*, thermal threshold, thermal variability, tipping point

## Abstract

Environmental variability is an inherent feature of natural systems which complicates predictions of species interactions. Primarily, the complexity in predicting the response of organisms to environmental fluctuations is in part because species' responses to abiotic factors are non‐linear, even in stable conditions. Temperature exerts a major control over phytoplankton growth and physiology, yet the influence of thermal fluctuations on growth and competition dynamics is largely unknown. To investigate the limits of coexistence in variable environments, stable mixed cultures with constant species abundance ratios of the marine diatoms, *Phaeodactylum tricornutum* and *Thalassiosira pseudonana,* were exposed to different temperature fluctuation regimes (*n* = 17) under high and low nitrogen (N) conditions. Here we demonstrate that phytoplankton exhibit substantial resilience to temperature variability. The time required to observe a shift in the species abundance ratio decreased with increasing fluctuations, but coexistence of the two model species under high N conditions was disrupted only when amplitudes of temperature fluctuation were high (±8.2°C). N limitation caused the thermal amplitude for disruption of species coexistence to become lower (±5.9°C). Furthermore, once stable conditions were reinstated, the two species differed in their ability to recover from temperature fluctuations. Our findings suggest that despite the expectation of unequal effect of fluctuations on different competitors, cycles in environmental conditions may reduce the rate of species replacement when amplitudes remain below a certain threshold. Beyond these thresholds, competitive exclusion could, however, be accelerated, suggesting that aquatic heatwaves and N availability status are likely to lead to abrupt and unpredictable restructuring of phytoplankton community composition.

## Graphical abstract

This figure is a generic representation of Jensen's inequality and scale transition theory, which are the cornerstone theoretical ideas explored in our manuscript.

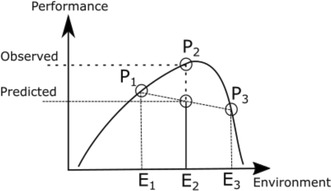

## 1 INTRODUCTION

Thermal fluctuations influence ecosystem structure and function. This is because temperature has a direct effect on metabolism and growth of aquatic organisms, and exerts control on the physical and chemical properties of water, including density, concentrations of dissolved gases, pH, and water column stratification/mixing due to solar heating and radiative cooling (e.g., Brierley & Kingsford, 2009; Constable et al., 2014; Grimaud et al., 2017; Kroeker et al., 2013; Poloczanska et al., 2016). The most common and profound changes in marine surface temperatures occur on diel and seasonal time scales (Doblin & van Sebille, 2016; Holbrook et al., 2020; Zaiss et al., 2021), but can also fluctuate within minutes when waters of different temperatures mix. The sea surface is a dynamic thermal environment influenced by changing seasons, weather events, horizontal advection, and ocean–atmosphere interactions (Deser et al., 2010; Shenoi et al., 2009). In the ocean, marine heatwaves (anomalous warming events) are becoming more frequent and intense (Oliver et al., 2018; Smale et al., 2019) leading to mortality, species range shifts, and community reconfigurations (Smale et al., 2019). In addition to thermal variability, nutrient concentration within marine ecosystems can fluctuate. This is especially the case for nitrogen which is often found to be a limiting nutrient for growth (Moore et al., 2013). However, N availability can fluctuate due to coastal upwelling, influx from river systems, atmospheric deposition, as well as varying rates of nitrogen fixation through N‐fixing organisms (Duce et al., 2008; Okin et al., 2011).

**FIGURE 1:**
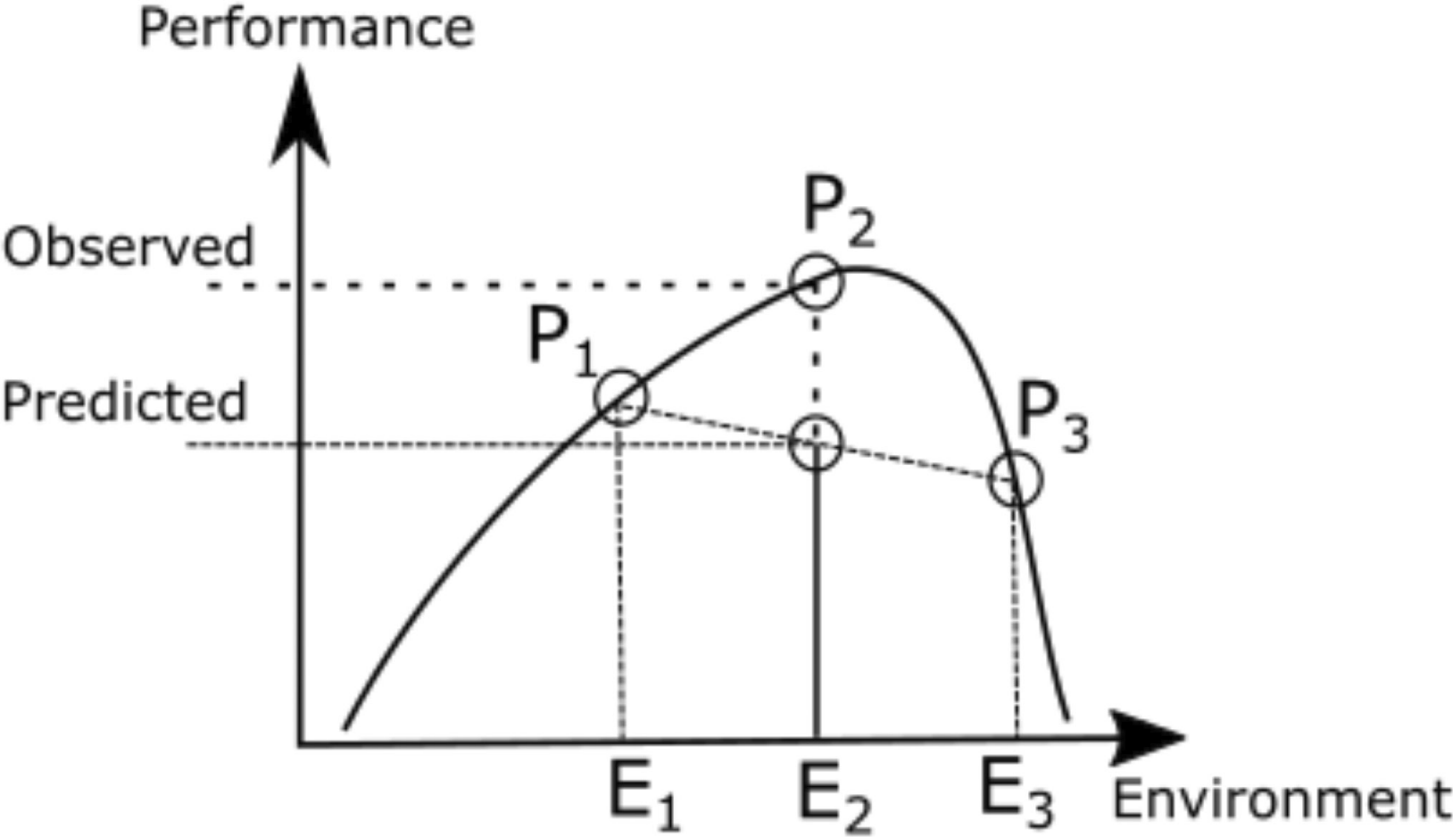
When performance of a species across an environmental gradient is non‐linear, performance P2 at the average environment E2, is not equal to the average value of performances P1 at environment E1 and P3 at environment E3. The mathematical generalization of this phenomenon has been coined Jensen's inequality (Denny, 2017), and when applied to ecology and ecophysiology, is referred to as scale transition theory (Chesson et al., 2005; Dowd et al., 2015).

In these fluctuating aquatic environments, phytoplankton is the dominant primary producers and form the base of the food web (Falkowski et al., 1998; Field et al., 1998). In response to the dynamic thermal environments in which they reside, marine phytoplankton possess a high degree of physiological flexibility (Boyd et al., 2013; Fey et al., 2021; Kremer et al., 2018). For instance, death rates of a marine diatom were shown to be dependent on acclimation temperatures and duration of heat stress (Baker & Geider, 2021), while cell pigment contents can change rapidly in response to differing light levels (Moore et al., 2006), or growth rates can acclimate and adapt quickly as a result of changes in CO_2_ or temperature levels (Padfield et al., 2015; Schaum et al., 2017). Typically, studies assessing the impact of temperature on phytoplankton examine responses under stable environmental conditions, and thus neglect both spatial and temporal environmental variability (Bernhardt et al., 2018). This poses a problem, however, as the per capita growth rate of a population is nonlinearly related to environmental variables and temporal variation in these variables (Bernhardt et al., 2018; Chesson et al., 2005; Dowd et al., 2015). Thus, the population growth rate in an environment where temperature (or some other variable of interest) fluctuates, differs from the growth rate in a constant environment with the same average temperature (Koussoroplis et al., 2017; Figure 1). Recently, this mechanism has been demonstrated in the laboratory, whereby oscillations in temperature generally result in lower growth rates (Bernhardt et al., 2018; Qu et al., 2019; Wang et al., 2019). In this way, thermal variability can influence competition between species and thus contribute to shaping community composition (Kling et al., 2020).

It has been suggested that competition in fluctuating environments is influenced by two mechanisms, the storage effect and relative nonlinear competitive variance (Chesson, 2000a, 2000b; Chesson, 2009). The storage effect refers to a mechanism by which temporal fluctuations create periods of time when resource availability, or escape from exposure to predators, benefits one of the competitors. During these beneficial periods, a competitor can bank ecological gains (e.g., higher population abundance or accumulation and storage of nutrients) that can then be drawn upon when the fluctuations favor the opposing competitor (Chesson, 2009). Alternatively, non‐linear effects in response to variances in competition can promote species coexistence by ensuring that populations will perform best at intermediate levels of competition. When a species' growth response is non‐linear to increasing levels of competition (i.e., growth is relatively unaffected at low levels of competition, and does not decrease any further at high levels of competition) then variability in the intensity of competition will allow a competitor to continue growing at optimum rates without negative effects from strong competitive pressure (Chesson, 2000b; Snyder & Chesson, 2004). The result of this effect might be that fluctuating environments could alter competitive dynamics up to a certain point, after which no further changes in the way two species compete are to be expected.

The effects of fluctuating nutrient supply (Chattopadhyay, 2007; Descamps‐Julien & Gonzalez, 2005; Gaedeke & Sommer, 1986; Sommer, 1984, 1995) or light regimes (Flöder & Burns, 2005; Litchman & Klausmeier, 2001) and their role in facilitating coexistence in experimental phytoplankton populations has been well documented. In contrast, the effect of temperature fluctuations is less well studied (Descamps‐Julien & Gonzalez, 2005). Given that thermal fluctuations are predicted to increase in intensity and become more frequent under climate change (Frölicher et al., 2018), it is important to understand the impacts of this variability on phytoplankton community dynamics. Furthermore, given that much of the ocean's primary productivity is limited by nitrogen (Duce et al., 2008; Moore et al., 2013), understanding potential interactive effects between different nutrient concentrations and thermal fluctuations is necessary. So far, studies examining interactions of alterations between multiple abiotic variables have focused mostly on whole community dynamics (e.g., Gerhard et al., 2019), but not on direct competition of specific species within a community.

In our previous study, we investigated competition dynamics between two marine diatoms, *Phaeodactylum tricornutum* and *Thalassiosira pseudonana*, across a stable temperature gradient and under two different nitrogen conditions (Siegel et al., 2020). *Phaeodactylum tricornutum* is a pleiomorphic diatom with the peculiarity that it does not seem, unlike other diatoms, to require silica for its growth. It is commonly considered a coastal species, and was first described in 1897. Since then it has been found in marine waters all over the world, ranging from the English Channel to the warm waters of Micronesia (De Martino et al., 2007). Although ubiquitously distributed, it is usually a rare species that does not dominate natural communities (personal communication with V. Chepurnov; De Martino et al., 2007). It is able to grow in a range of salinities, from brackish rock pools to marine waters, and has even been cultivated in freshwater (De Martino et al., 2007). In contrast, *T. pseudonana* requires silica for growth and is therefore more representative of diatoms from a morphological point of view. It has been found in fresh and saline waters across the world, ranging from open‐ocean environments to coastal zones and lakes (Alverson et al., 2011). *Phaeodactylum tricornutum* and *T. pseudonana* could therefore theoretically compete against each other in natural communities, and have been found to do so in mass culture pools derived from waters coming off the coast of the North‐Eastern United States (Goldman & Ryther, 1976).

The aim of the previous study described in Siegel et al. (2020) was to understand to what extent the competitive outcomes between this species pair depend on temperature and nitrogen availability under stable (steady‐state) conditions—that is the absence of environmental fluctuations. The study showed that the competitive outcome, that is, which species will survive and outgrow the other, depended on the environmental temperature. On average, *P. tricornutum* ‘won’ competitions across lower temperatures, whereas *T. pseudonana* was the better competitor at warmer temperatures. Outcomes of competition were also influenced by the amount of nitrogen present in the growth medium. When nitrogen was abundantly present, *P. tricornutum* won competitions up to 18.8°C, whereas when N stocks were depleted, it won competitions up to 25.3°C. Thus nitrogen limitation enabled *P. tricornutum* to win competitions across a greater temperature range than under N‐replete conditions. The temperatures at which competitive advantages switched over from one species to another were identified as temperature points of potential stable coexistence between the two species. We found that coexistence was possible at 18.8 ± 1.2°C in nutrient replete high N conditions, and at 25.3 ± 0.6°C in nutrient‐limited low N conditions.

The present study builds upon our previous investigation on competition dynamics between *P. tricornutum* and *T. pseudonana*. The aim was to elucidate the role of environmental variations in determining outcomes of competition in response to temperature fluctuations under both nutrient‐replete and nitrogen‐limited conditions. We hypothesized that stable coexistence between the two diatoms could be achieved in a fluctuating thermal environment as long as the mean temperature of stable coexistence remained constant. We, however, presumed that just like under stable conditions, there could exist interactive effects between temperature and N concentration that influence the coexistence of the two species.

To elucidate these hypotheses, we first confirmed the coexistence temperatures under stable thermal conditions identified in our previous paper (18.8 ± 1.2°C in high N conditions, and at 25.3 ± 0.6°C in low N conditions). We then went on to investigate whether uniform fluctuations with varying amplitudes around the temperatures of stable coexistence would disrupt the coexistence of these two species. We concluded our investigation on thermal fluctuations by investigating if potential mixed population stability would re‐establish itself after a period of temperature fluctuations when temperature was stabilized once again. Because differences in the abundances of initial community members were found to play a role in determining final community structure in nature (Sommer, 1983), we additionally investigated whether this would be the case in a simplified and controlled system such as ours. Finally, we used data from Siegel et al. (2020) and compared them to the findings from the current study to explore competitive exclusion between the two diatoms in different thermal‐nutrient regimes.

## 2 MATERIALS AND METHODS

Stock culture upkeep, and acclimation to experimental conditions of *P. tricornutum* (CCMP 2561) and *T. pseudonana* (CCMP 1335), as well as monitoring of biomass via fast repetition rate fluorometry (FRRf) was performed as in Siegel et al. (2020), with peak excitation at 435 nm and fluorescence emission being measured at 680 nm (with a 25 nm bandwidth). The measuring protocol was set to 24 sequences per acquisition with a 100 ms sequence interval and a 20 s acquisition pitch. In vivo chlorophyll *a* minimum fluorescence (*F*_*0*_), and the ratio of variable to maximum fluorescence (*F*_*v*_ */F*_*m*_) from FRRf were used as proxies for biomass and photosynthetic efficiency, respectively. Briefly, cultures were acclimated to high N exponential growth and low N semi‐continuous growth 2 weeks prior to the onset of the competition experiments. After the acclimation period, the experiments were conducted as described below.

Data on thermal performance curves (TPCs) of the two species were collected in the previous publication and can be re‐visited alongside the cardinal temperatures of the two species in the Supplementary Material to this publication (Figures S1 and S2).

### 2.1 Competition experiments

**FIGURE 2:**
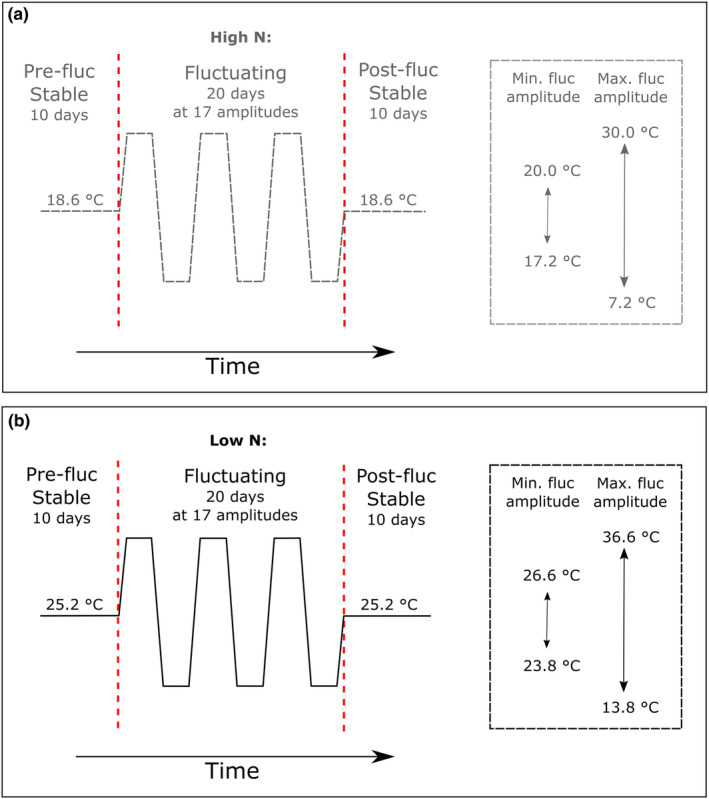
Schematic timeline of the fluctuation experiments under (a) high N conditions and (b) low N conditions. Each experiment consisted of a 10‐day pre‐fluctuation phase in which *Phaeodactylum tricornutum* and *Thalassiosira pseudonana* were cultured as mixed populations under high N and low N conditions at the temperature of stable coexistence, determined experimentally as 18.6°C and 25.2°C, respectively. After this initial period of thermally constant conditions, the temperature was fluctuated with differing amplitudes (*n* = 17) for 20 days, consisting of 10 “cool” (troughs) and 10 “warm” (peaks), each of 24 h duration. The 17 thermal amplitudes ranged between ±1.4°C in the ‘weakest’ treatment level and ±11.4°C for the ‘strongest’ treatment level. The temperature transition between each trough and peak was relatively sharp, as the “new” temperature stabilized within 1 h. Following the fluctuation phase, experimental conditions were re‐stabilized to the (constant) temperature of stable coexistence for a further 10 days. This post‐fluctuation stage of the experiment aimed to mimic a “recovery” phase to investigate whether any disruption of species coexistence could be reinstated once stable conditions were restored.

The first phase of the experiment, herein referred to as the “pre‐fluctuation phase,” consisted of an acclimation period for monocultures to adjust to the experimental set‐up and a constant temperature phase to allow mixed cultures to achieve stable coexistence. In the second phase of the experiment, herein termed the “fluctuation phase,” the mixed cultures were exposed to a gradient of fluctuation amplitudes before being returned to a recovery period, termed “post‐fluctuation phase.” A schematic representation of the experimental chronology is presented in Figure 2. The purpose of this experimental design was to confirm that the temperature points (18.8 ± 1.2°C in high N conditions, and at 25.3 ± 0.6°C in low N conditions) which were previously identified in Siegel et al. (2020) to sustain coexistence between the two species, would allow cultivation of the two species as stable mixed communities without one species outcompeting the other (“pre‐fluctuation phase”). In the “fluctuation phase,” we then manipulated the thermal conditions to elucidate whether fluctuations of different thermal amplitudes around the points of stable coexistence would disrupt the potentially stable mixed cultures that were established in the “pre‐fluctuation phase.” During the “post‐fluctuation phase,” temperatures were returned to the initial temperature points within each nitrogen treatment. This was done to investigate whether species abundance ratios would return to being stable over time or whether thermal fluctuations would cause potential longer term disruption of competitive stability after they had ceased.

#### 2.1.1 High N competition experiment

For the high N scenario, pre‐acclimation was achieved by incubating monocultures of each diatom species (*n* = 34; 5 mL volume) for 2 days in a tube‐based temperature‐gradient‐thermoblock at 18.6°C and 150 ± 15 μmol m^−2^ s^−1^ photosynthetic photon flux density (PPFD). Our previous experiments have shown that this temperature facilitates coexistence in a stable equilibrium under high N conditions (Siegel et al., 2020).

After 2 days of acclimation in the temperature‐gradient‐thermoblock, the monocultures of *P. tricornutum* and *T. pseudonana* were combined to form “mixed cultures”. To determine whether the competition outcome was influenced by the initial species abundances, the experiment was conducted using three different starting relative cell abundance ratios of 1:1 (*n* = 24), 1:2 (*n* = 24), and 2:1 (*n* = 20). The total amount of cells within each starting culture approximated 100,000 cells mL^−1^. Mixed cultures were maintained in exponential growth by re‐diluting when necessary, as described in Siegel et al. (2020).

To confirm that stable coexistence was achieved at 18.6°C, the cell abundance ratios of the mixed cultures were monitored daily for 10 days. To calculate cell abundance ratios, an aliquot (200 μL) was harvested each day after FRRf measurements of *F*_*0*_ and *F*_*v*_ */F*_*m*_, fixed in 1%‐Lugol's solution and stored in 96‐well plates until analysis (within 24 h). In order to maintain a constant culture volume, the 200 μL volume removed from each culture was replenished with the same volume of fresh F/2 medium. The *P. tricornutum*:*T. pseudonana* cell abundance ratio was calculated from 400 cells that were counted using an Olympus inverted microscope at 200× magnification.

For the fluctuation phase of the experiment (Day 11 to Day 30), temperature fluctuations were imposed by heating one end of the temperature‐gradient‐thermoblock, while the other end remained unchanged; this strategy created a thermal gradient from 30.0°C at the left (hot end) to 20.0°C at the right (cold end) of the block. After 24 h, the heated end of the thermoblock was cooled, reversing the thermal gradient from a low of 7.2°C at the left of the block to a high of 17.2°C at the right end of the block. This experimental design resulted in 17 levels of thermal amplitudes, with a minimum peak‐to‐trough amplitude ranging from ±1.4°C (with a peak and trough temperature of 20 and 17.2 °C, respectively, at the right end of the block) to ±11.4°C (with a peak and trough temperature of 30 and 7.2°C, respectively, at the left end of the block) relative to the temperature of stable coexistence at 18.6°C. Samples for determination of species abundance ratios were taken daily, but counts on abundance ratios over time during the fluctuation period were performed on average every 2 days.

To determine whether the initial temperature exposure had an impact on competition outcome (i.e., treatment starting on a peak or a trough), half of the cultures were started on a peak and the other half were transferred to a water bath that was also set to 18.6°C and 150 ± 15 μmol m^−2^ s^−1^ PPFD. After 24 h these cultures were re‐introduced to the temperature‐gradient‐thermoblock and exposed to the temperature fluctuations starting in a trough. Similarly, at the end of the fluctuation phase, cultures initiated on a peak amplitude were transferred to the external water bath, so that the cultures beginning on the trough were exposed to an equal number of peaks and troughs.

In the post‐fluctuation phase, we investigated whether the prolonged period of thermal variability prevented the re‐establishment of stable coexistence. In this phase of the experiment, after assay cultures were exposed to fluctuating temperatures for 20 days (10 peaks and 10 troughs), the temperature of the block was returned to 18.6°C and cell abundance ratios were monitored for a further 10 days and sampled on a daily basis. This experimental timeline resulted meant that the cultures starting their fluctuation period on a trough had 11 days of stable pre‐fluctuation conditions, while those starting on a peak had 11 days of stable post‐fluctuation conditions.

#### 2.1.2 Low N competition experiment

Under low N conditions, monocultures of *P. tricornutum* and *T. pseudonana* (*n* = 34 per species) were transferred from stock cultures into experimental conditions and grown until onset of N depletion at 150 ± 15 μmol m^−2^ s^−1^ PPFD and 25.2°C (determined by pre‐experiments to be the temperature of stable coexistence; Siegel et al., 2020). In each tube with 5 mL culture volume, N drawdown was monitored daily by removing 20% of the culture and replenished with fresh low N amended F/2 medium (55 μM NO_3_^−^). Cultures were allowed to grow until N‐depleted stationary growth phase. The onset of N depletion was determined via FRRf as the point at which the fluorescence signal (*F*_*0*_) did not increase between two consecutive days and the sum of nitrate plus nitrite (NO_x_) was below the detection limit of 0.5 μM of the method (Schnetger & Lehners, 2014). Once N depletion was confirmed, which was the case after four culturing days, mixed cultures were established. Similar to the high N mixed populations, the monocultures were mixed together so that starting *P. tricornutum:T. pseudonana* cell abundance ratios were either 1:1 (*n* = 24), 2:1 (*n* = 24) or 1:2 (*n* = 20). Mixed cultures were grown semi‐continuously by removing 1.0 mL daily after FRRf measurements were made, and replenishing with 1.0 mL of low N medium to maintain an assay volume of 5.0 mL. By removing 20% of the culture medium and replenishing the remaining 80% with fresh medium, the daily growth rate imposed by this dilution was ln (5/4) day^−1^ = 0.22 day^−1^ after the stationary phase had been reached in the mixed cultures. From the removed 1.0 mL sample volume, 200 μL subsamples were then fixed in 1%‐Lugol's solution for cell abundance counts.

To ensure the two nutrient scenarios were comparable, a similar experimental chronology was adopted (Figure 2). Specifically, cultures were kept at the temperature of stable coexistence for 10 days before being subjected to 20 days of temperature fluctuations. The amplitudes were the same as during the high N experiment (17 fluctuation amplitudes ranging between ±1.4°C and ±11.4°C); however, the mean was set to 25.2°C; the temperature of stable coexistence under low N conditions (Siegel et al., 2020). Following exposure to 10 peaks and troughs, the temperature was once again stabilized at 25.2°C for a further 10 days. Like in the high N scenario, half of the cultures were held back for another day at the beginning and end of fluctuations at the temperature of stable coexistence, so that the influence of a peak and trough start and end could once again be investigated.

### 2.2 Statistical analysis

#### 2.2.1 Observed competition in fluctuating environments

Observed competition coefficients (*C*_*i*_ in Equation 1 below) of the high N and low N scenarios were determined by calculating the slope of the natural logarithm of the *P. tricornutum:T. pseudonana* cell abundance ratios over time for each fluctuation amplitude (*F*_i_) in the “pre‐fluctuation,” “fluctuation,” and “post‐fluctuation” phases of the experiment.

(1) $$\ln\;\left(P.\;\text{tricornutum}:T.\;\text{pseudonana}\right)=C_{0}+C_{i}∙t$$

where *C*_*0*_ is the value of the ln(*P. tricornutum:T. pseudonana*) ratio at the start of the phase, *t* is time in days, and the competition coefficient (designated *C*_*i*_) is the slope of the relationship. A positive slope indicates that the relative abundance of *P. tricornutum* increased over time in mixed cultures. In contrast, a negative slope results from an increase in relative abundance of *T. pseudonana* through time. As such, the positive competition coefficients are indicative of *P. tricornutum* winning the competition, whereas negative competition coefficients are indicative of *T. pseudonana* winning.

We did not observe a difference in the progression of species abundance ratios over time that related to the cultures starting on a trough or peak fluctuation (Figures S5 and S6). Therefore, the data were pooled for analysis.

#### 2.2.2 Stability of mixed cultures

Mixed cultures were considered stable if the slope of Equation 1 was not significantly different from zero, indicating that there was no change in relative abundance of the two species through time. This was the case for all of the cultures in the pre‐fluctuation stage (Table S1).

To identify whether fluctuation amplitude influenced mixed culture dynamics, we used piecewise regression to identify the fluctuation amplitude (°C) at which the mixed diatom population became unstable, that is, the fluctuation amplitude at which the competition coefficient changes abruptly from approximately zero. Piecewise regression techniques have previously been used as a statistical method to model ecological thresholds (Toms & Lesperance, 2003). These models are also known as broken‐stick models, and can be described as follows:

The simplest relationship is a linear model that does not have a breakpoint (Equation 2).

(2) $$\;C_{i}=\beta_{0}+\beta_{1}F_{i}+e_{i}\;$$

Where *C*_*i*_ is the competition coefficient for the *i*th fluctuation amplitude, *F*_*i*_ is the corresponding fluctuation amplitude, β_*0*_ is the intercept at *F*_*i*_ = 0, β_*1*_ is the slope of the linear relationship, and *e*_*i*_ the residual error.

Where there is one break point, the model becomes:

(3) $$\;C_{i}=\left\{\begin{array}{c} \beta_{0}+\beta_{1}F_{i}+e_{i}\;\text{for}\;F_{i}\leq \alpha \\ \beta_{0}+\beta_{1}F_{i}+\beta_{2}\left(F_{i}-\alpha \right)+e_{i}\;\text{for}\;F_{i}>\alpha \end{array}\right.$$

where *α* is the breakpoint (i.e., threshold) at which the slope changes from β_*1*_ to β_*1*_ + β_*2*_, and hence β_*2*_ is the difference in slopes.

Similarly, we can estimate two breakpoints that incorporate three linear segments as following:

(4) $$\;C_{i}=\left\{\begin{array}{c} \beta_{0}+\beta_{1}F_{i}+e_{i}\\ \beta_{0}+\beta_{1}F_{i}+\beta_{2}\left(F_{i}-\alpha \right)+e_{i}\;\\ \beta_{0}+\beta_{1}F_{i}+\beta_{2}\left(F_{i}-\alpha \right)+\beta_{3}\left(F_{i}-\alpha_{2}\right)+e_{i}\; \end{array}\;\begin{array}{c} \text{for}\;F_{i}\leq \alpha_{1}\\ \;\text{for}\;\alpha_{1}<F_{i}\leq \alpha_{2}\\ \text{for}\;F_{i}>\alpha_{2} \end{array}\right.$$

where *α*_*1*_ and *α*_*2*_ are the first and second breakpoint, respectively, and the slopes of the lines are β_*1*_, β_*1*_ + β_*2*_, and β_*1*_ + β_*2*_ + β_*3*_. Therefore, β_*3*_ is the difference between the second and third slopes, respectively.

Piecewise regression analysis was conducted using the R package “segmented” (Muggeo, 2008). The significance of this breakpoint was assessed using sequential hypothesis testing via the Score test.

#### 2.2.3 Time scales for changes in species abundance ratios

Since observed competition coefficients were calculated as the linear regression of the natural logarithm of species abundance ratio changes over time, it was possible to calculate the time required (days) for the cell abundance ratio to change by 50% (using Equation [5]). This value was used as a further indicator for temporal stability of mixed communities.

(5) $$50\%\text{change in abundance ratio}=\frac{\ln\left(0.5\right)}{ǀSǀ}$$

where ǀ*S*ǀ is the absolute value of the mean competition coefficient.

Time scales were also calculated with the data for competition at different stable assay temperatures (*n* = 15 for high N conditions and *n* = 14 for low N conditions) reported in Siegel et al. (2020), allowing a comparison between the times required to reach a 50% change in species abundance ratios between stable and fluctuating thermal environments.

All data analysis and calculations mentioned above were carried out with the statistical software R (version 3.3.1; R Core Team, 2016).

## 3 RESULTS

Different starting cell abundance ratios of 1:2, 1:1, or 2:1 (*P. tricornutum:T. pseudonana*) did not influence mixed culture stability during the pre‐fluctuation period (Figures S3 & S4). Consequently, the data from all starting ratios were pooled for the analysis.

### 3.1 Analysis of species stability in mixed communities

**FIGURE 3:**
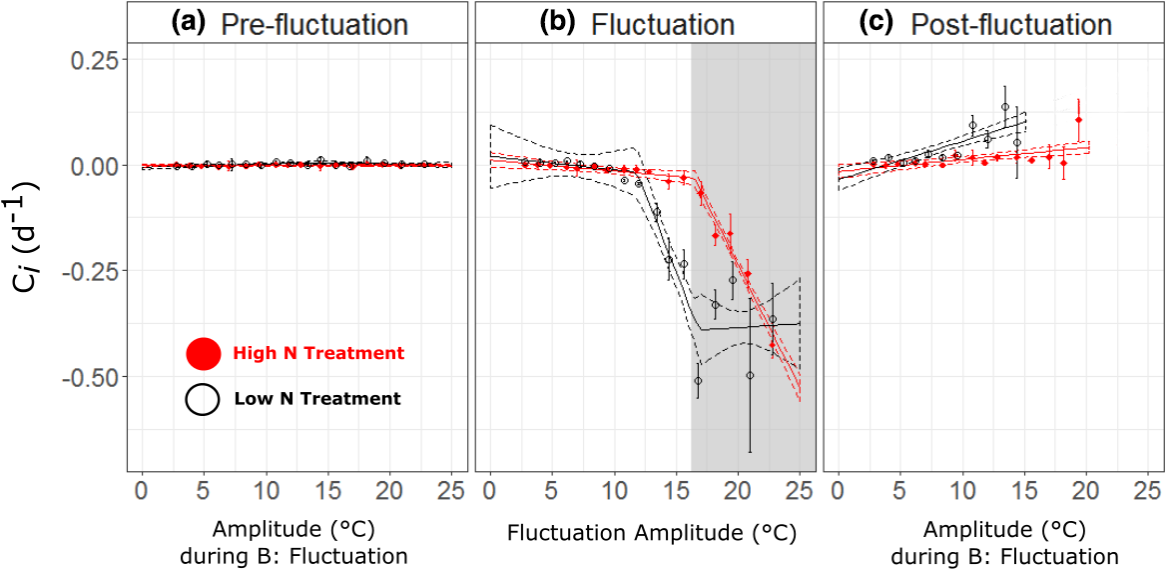
Relationship between competition coefficients and temperature divergence from the temperature of coexistence during (a) the pre‐fluctuation, (b) fluctuation, and (c) post‐fluctuation experimental stages, under high N (red), and low N (black) conditions. Positive values indicate that *Phaeodactylum tricornutum* is the winner and negative values that *Thalassiosira pseudonana* is the winner. Symbols are the mean observed competition coefficients of biological replicates with error bars indicating 95%‐confidence intervals (CI). Solid lines represent regression analyses (Equations [2], [3] & [4]), with dotted lines corresponding to the 95% CI. Note that while amplitudes (divergence from mean) are consistent between N treatments, the mean temperatures differ due to differences in the temperature of stable coexistence (18.6°C and 25.2°C, for high and low N, respectively). In (B), gray shaded area indicates fluctuation treatments of low N in which *P. tricornutum* died following exposure to one warm 24 h period (based on *Fv*/*Fm* measurements of monocultures). In contrast, *T. pseudonana* survived in mixed cultures for the remainder of the experiment (Appendix S1; “Limitations of the study”).

Regression analyses were used to analyze the relationship of competition coefficient to thermal amplitude collected during (a) the pre‐fluctuation, (b) fluctuation, and (c) post‐fluctuation experimental stages, under both low and high N conditions (Figure 3).

During the initial pre‐fluctuation period, the abundance ratios between the two diatom species showed little change through time, as the slope of the regression for high N and low N treatments was not significantly different from zero (Table S1), meaning within each N treatment, the mixed diatom populations were in stable coexistence before the thermal fluctuation stage of the experiment began (Figure 3a).

In contrast, during the fluctuation phase (under both low and high N conditions), we identified significant break points in the broken‐stick model, meaning there was a threshold in the fluctuation amplitude, such that cultures became unstable once this threshold was exceeded (i.e., the competition coefficient differed from zero). Under high N conditions, the broken‐stick model identified a single breakpoint at a divergence from the coexistence temperature at a fluctuation amplitude of ±8.2 °C from the temperature of stable coexistence (95% CI; 8.0, 8.5), whereby at fluctuations above this threshold the competition coefficient shifted in favor of *T. pseudonana* (*β*_2_ = −0.111; Table 1). However, under low N conditions, we identified the first breakpoint (α_1_) at a significantly lower fluctuation amplitude of ±5.9°C from the temperature of stable coexistence (95% CI; 5.3, 6.5), meaning the competition coefficient shifted in favor of *T. pseudonana* at lower magnitudes of variability with stronger coefficient (*β*_2_ = −0.144; Table 1). The second breakpoint (α_2_), identified at a divergence from the mean of ±8.4°C (95% CI; 7.7, 9.1) corresponded with the thermal threshold at which *P. tricornutum* monocultures did not survive following 24 h of exposure to the high‐temperature extreme (Figure 3b, gray shaded area). Hence, the highly negative competition coefficient values in this region are due to *T. pseudonana* being the only “survivor.”

**TABLE 1:** Parameters of the (intercept‐only) linear model (Equation 2), and broken‐stick models (Equations 3 and 4) for the relationship between the competition coefficient and amplitude of temperature fluctuation (°C).

Stage	N	Model	Equation	*β*_*0*_	*β*_*1*_	*β*_*2*_	*β*_*3*_	*α*_1_	*α*_2_	*R* ^2^	Adj. *R* ^2^	AIC	BIC
Pre	Low	Intercept‐only	2	0.0014 ns						‐	‐	−485.1	−480.7
	High	Intercept‐only	2	−0.0015 *						‐	‐	−522.8	−518.4
Fluctuating	Low	Broken stick	4	0.0197 ns	−0.0062 ns	−0.1441***	0.1539***	5.9	8.4	0.85	0.84	−152.2	−136.7
	High	Broken stick	3	0.0109 ns	−0.005***	−0.1112***		8.2		0.96	0.96	−304.9	−293.8
Post	Low	Intercept‐only	2	0.0395 ***						‐	‐	−244.3	−240.1
	High	Intercept‐only	2	−0.0156 ns	0.0056***					0.23	0.21	−257.6	−251.3

*Note*: ns indicates non‐significance at *p* ≥ 0.05. * Indicates significance at *p* < 0.05, ** Indicates significance at *p* < 0.01, *** Indicates significance at *p* < 0.001, AIC is Akaike's information criterion; BIC is the Schwarz–Bayesian information criterion.

For brevity, only the “best” fitting models (based on AIC scores) are shown here; see Table S7 for extended model results. Competition coefficients were calculated as the slopes of regression of the change in species abundance versus time (Equation 1) for pre‐fluctuating (Pre), fluctuating, and post‐fluctuation (Post) stages of the experiment under both low and high N conditions. In the regression models *β*_*0*_ is the intercept (i.e., the competition coefficient when temperature fluctuation amplitude (±°C) equals zero), *α*_*1*_ is the breakpoint temperature fluctuation amplitude (±°C) at which the slope of the regression changes from *β*_*1*_ to *β*_*1*_ + *β*_*2*_ and hence *β*_*2*_ is the difference in slopes on either side of the threshold *α*_*1*_. The linear model does not have a breakpoint. Where two breakpoints were identified, *α*_*2*_ is the second breakpoint temperature fluctuation amplitude (±°C), at which the slope changes from *β*_*1*_ *+ β*_*2*_ *to β*_*1*_ *+ β*_*2*_+ *β*_*3*_, where *β*_*3*_ is the difference in slopes either side of the second threshold *α*_*2*_. In the case where no breakpoint was identified, or the broken stick model was less parsimonious, linear regressions were used.

Following 20 days of an imposed fluctuating regime and after thermal conditions were returned to a constant temperature (i.e., fluctuation amplitude equals zero), cultures did not exhibit the same behavior as during pre‐fluctuation conditions. The post‐fluctuation analysis was performed on the cultures where both species survived (high N conditions; *n* = 60, low N conditions; *n* = 43). Under high N conditions, we identified a strong and significant positive relationship between competition coefficient and the prior fluctuation amplitude (*β*_1_ = 0.0156; *p* < 0.001) meaning that *P. tricornutum* became more competitive when the fluctuations ceased. Notably, this competition advantage strengthened further (*β*_1_ = 0.0180; *p* < 0.001) under low N conditions.

Together these results suggest that (i) thermal variability has the potential to disrupt stable coexistence, (ii) N limitation can reduce the fluctuation amplitude at which this coexistence becomes unstable, and (iii) coexistence may not necessarily re‐establish once conditions return to constant temperatures; resulting in unpredictable outcomes of competition in a formerly stable mixed population that encountered a fluctuating environment.

### 3.2 Competitive exclusions are an interplay between thermal variability and nutrient regime

**FIGURE 4:**
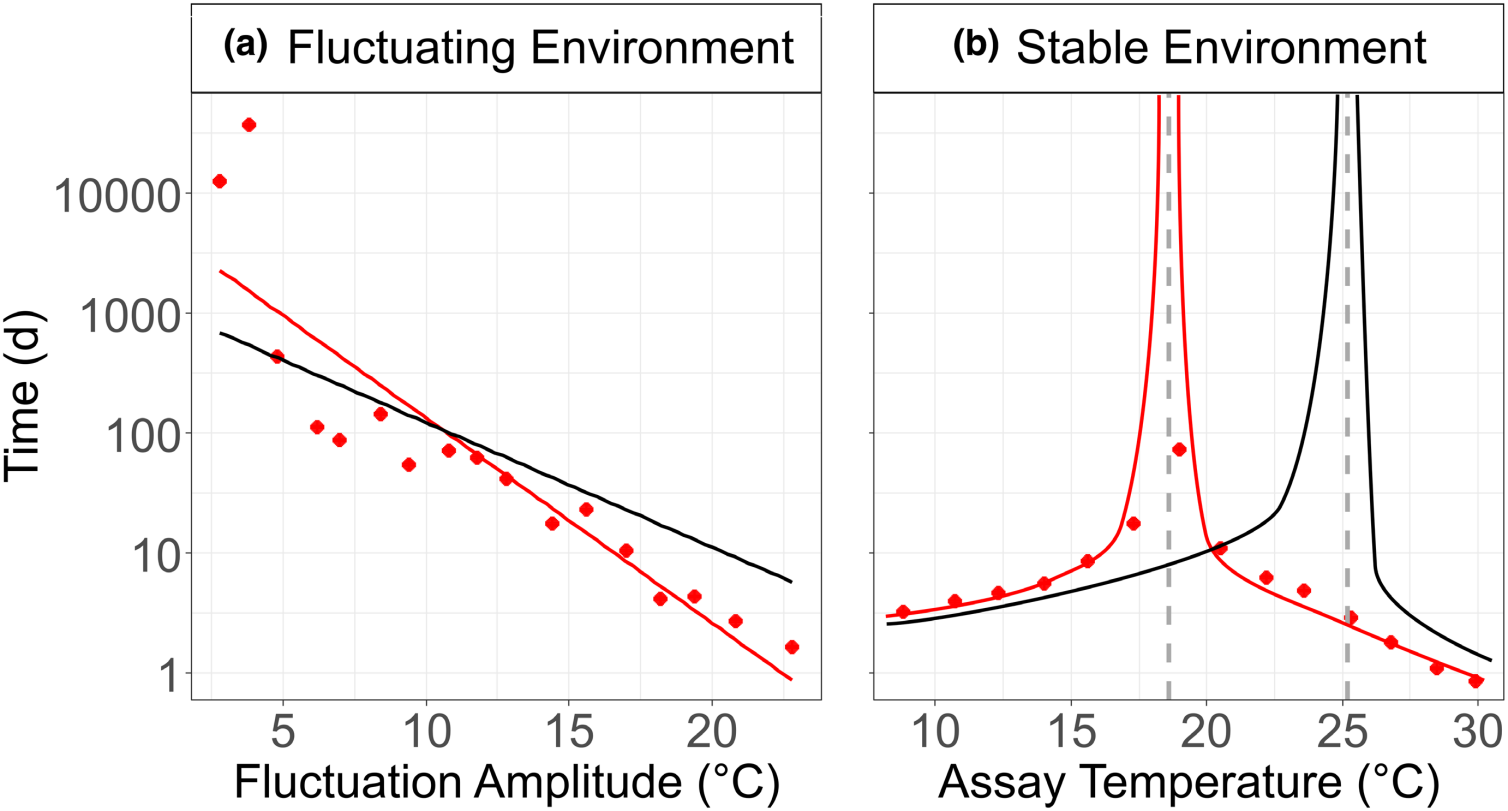
Time (days) taken to reach a 50% change in species abundance ratios in (a) fluctuating, and (b) stable temperature environments for high N (red) and low N (black) mixed cultures. Note that *y*‐axis is presented on a log10 scale to show that the time it takes encompasses periods from days and weeks to years. In (a), lines are from fitting linear models to the data. In (b), the lines are a visual aid to demonstrate that as the assay temperature approaches the temperature of stable coexistence, time approaches infinity. Gray dashed vertical lines visualize the temperature of stable coexistence in a stable thermal environment (18.6°C for high N and 25.2°C for low N mixed cultures).

In our second approach to assess the effect of thermal fluctuations on species displacement, we combined the results from this study with those collected in Siegel et al. (2020). As fluctuation amplitude increased, we observed a reduction in the time taken to reach a 50% change in abundance ratio (linear regression; Figure 4a). In contrast, by applying competition coefficients from our previous work, we show that in a stable thermal environment (fluctuation amplitude equals zero), the relationship is non‐linear; as the temperature approaches the point of stable coexistence, time approaches infinity (Figure 4b).

A further marked difference between stable and fluctuating thermal environments is the difference in how quickly a competitor was displaced in a mixed population. Under constant temperatures, and in both nutrient regimes, a 50% change in species abundance ratios occurred within 10 days following a shift of temperature by ±1.5°C from the temperature of stable coexistence. Whereas in fluctuating regimes similar levels of species displacement over similar time frames (i.e., 10 days) required temperature amplitudes to exceed ±8.5°C Hence, in a fluctuating thermal environment the time taken for a species to displace another is lengthened and thermal variability therefore favors the “losing” species.

Finally, we find that nitrogen limitation in conjunction with thermal variability can further decelerate the displacement of the “losing” species. Our results show that the time taken for one species to outcompete the other was dependent on a combination of the nutrient scenario (low or high N) and fluctuation amplitude (Figure 4a). Regimes characterized by low N and high fluctuation amplitudes were more advantageous to the displaced species. Specifically, when fluctuating amplitudes were greater than or equal to ±2.5°C, N depletion increased the time required for species displacement to occur via competition (i.e., time taken to reach 50% change in abundance ratio was greater than under high‐N conditions). In contrast, under low thermal amplitudes (less than ±2.5°C), displacement occurred more rapidly than under high N conditions, that is, shorter time taken to reach 50% change in abundance ratio. Together our results show that conditions are more advantageous to the “winning” species when nutrients are replete and temperature fluctuations are small as they are able to displace the “losing” species more quickly. However, under greater thermal variability when nutrients become depleted and growth rates slow, the “losing” species is displaced more slowly. Consequently, the degree of temperature disturbance and nutrient regime imposes an interesting interplay on species competition.

## 4 DISCUSSION

While N limitation decreases thermal tolerance toward warm temperatures (Thomas et al., 2017) and alters competition dynamics in marine phytoplankton (Siegel et al., 2020), this study is the first to examine the interaction of thermal variability and nutrient availability on direct competition and stable coexistence of phytoplankton. We show that thermal variability disrupts species coexistence, and that weaker disturbances are required to unravel this stability when nitrogen is limiting. Coexistence between the diatoms *P. tricornutum* and *T. pseudonana* was maintained during conditions of high thermal variability (amplitudes up to about ±8 °C under nutrient replete conditions; about ±6°C under N limitation). The decrease in the amplitude of thermal fluctuation that allows coexistence of these two species in N‐limited conditions can be attributed to the shift in the stable coexistence temperature closer to the upper thermal tolerance limit. Additionally, while stable coexistence can be maintained in N‐limited environments during periods of low thermal variability, differences in the way species recover from these events mean that competitive outcomes can be delayed until the system returns to “normal,” more constant conditions. Together our results reveal that N limitation reduces the magnitude of thermal variability over which stable species coexistence occurs and impacts the ability of the less competitive species to recover—exacerbating species displacement. Thus, our findings have implications for species competition in the warmer, more thermally‐variable and N‐limited oceans predicted in the future (Raven & Beardall, 2021). In addition, our competition experiments yield important insights into the mechanisms that underlie species distributions and help to provide evidence toward the resolution of the “Paradox of the plankton” (Hutchinson, 1961).

Here we show that thermal variability can provide a refuge for the species that would otherwise be outcompeted if thermal conditions were stable. Only at the temperature point of stable coexistence would a constant temperature environment be more beneficial for species coexistence than a fluctuating environment. Indeed, periodically alternating between brief periods of warm and cool phases facilitated continued coexistence between the two diatom species for much longer than observed at constant temperatures. As temperature fluctuated, the hot peaks created favorable growth conditions for one species (and the cold troughs for the other), such that each species was able to “store gains” under favorable temperatures allowing it to survive unfavorable periods (Chesson, 2009). In natural environments, such thermal refuges may delay or prevent species displacement if the population can be replenished via migration or mixing. Likewise, when growth rates of the superior competitor are slowed, they are more susceptible to grazing pressure or being “washed away.” If the exclusion rate of one species is low and the immigration rate is high, disadvantageous environmental conditions can be counterbalanced, as immigration increases spatiotemporal resilience (Gonzalez & Holt, 2002). Consequently, fluctuations in abiotic variables such as temperature can foster ecological resilience of a diverse community of competitors and help to explain the high degree of species diversity observed in nature.

Climate change simultaneously alters many environmental factors that regulate phytoplankton ecophysiology (Boyd et al., 2010). These factors include temperature and nutrient concentrations, and due to their control on primary production, their influence can be observed at regional and global scales (Anderson et al., 2022; Behrenfeld et al., 2005; Falkowski et al., 1998; Moore et al., 2013; Salk et al., 2021). Here, we report a synergistic interplay between nutrient availability and thermal variability with nitrogen limitation diminishing the competitiveness of the “winning” species and lengthening the time taken to displace the “losing” species, thereby broadening the haven that temperature fluctuations provide. By exerting a proximate control on growth and photosynthesis, nutrient limitation can reduce the magnitude of the temperature dependence of growth (Bestion et al., 2018; Edwards et al., 2016; Marañón et al., 2018). Therefore, species that can persist under low nitrogen environments, for example, in the tropical open‐ocean or estuaries and coastal environments during less productive seasons (Dijkstra et al., 2019; Silkin et al., 2019; Varol, 2019), may maintain their competitive advantage under thermally variable conditions (Bestion et al., 2021; Descamps‐Julien & Gonzalez, 2005; Tilman, 1977). As such, the synergistic interplay between nitrogen availability and thermal variability means that while the growth rates of all species are reduced, the dominant species are affected disproportionately. The implications being that although primary production may be reduced in the short‐term (hours to days), high species richness can be maintained in the long term (weeks to months), resulting in a community more resilient to environmental extremes.

In aquatic environments, diurnal fluctuations in surface temperatures commonly reach ±1.5°C, and in extreme cases up to ±5°C (Karagali et al., 2012; Kawai & Wada, 2007; Shenoi et al., 2009), with inter‐seasonal or inter‐annual variations being even more pronounced (Dowd et al., 2015). The stable coexistence of *P. tricornutum* and *T. pseudonana* observed here was maintained during thermal fluctuations that are typical in natural environments such as small lakes (less than 3 km^2^), where the average daily surface temperature can vary between 4 and 7°C (Woolway et al., 2016). Indeed, our lower fluctuation amplitudes are in line with such variability, supporting the view that variable environments can provide thermal refuges, and may explain why high phytoplankton species diversity can be observed in lakes (Sommer, 1984). Importantly, however, we find that periods of high thermal variability can result in unpredictable competition outcomes once more stable “normal” conditions return. Specifically, in scenarios where thermal fluctuations exceeded ±9 °C and constant conditions were reinstated, we observed competitive exclusion at temperatures where coexistence was previously observed. Thermal variability of this magnitude was for instance observed in coastal environments in which both *P. tricornutum* and *T. pseudonana* are found (Alverson et al., 2011; De Martino et al., 2007), such as tidal pools in southwest England, where summer daily tide‐out thermal fluctuation amplitudes have been recorded at ±9°C (between 12–30°C; Kleiner et al., 2022). Moreover, the “losing” species during conditions of large fluctuations became the “winning” species during the recovery phase, and notably this competition advantage was strengthened in a low N environment. Hence, species‐specific differences in the ability to recover from environmental variability can influence competition outcomes in the longer term. Consequently, monitoring species dynamics once the disturbance has passed, that is, during the system's “recovery” phase, can provide a more accurate assessment of the ongoing implications of variable environments on species competition.

Climate change is predicted to make the ocean surface waters warmer and more N‐limited, and temperature fluctuations are likely to be more frequent and extreme (Boyd et al., 2016; Frölicher et al., 2018). Likewise, freshwater environments are predicted to be subjected to heatwaves that are more pronounced and more persistent (Schmidt et al., 2018), and lakes are predicted to experience alterations in their N concentrations that can tip in either direction: Depending on the system under observation, nitrogen concentrations could increase due to more anthropogenic inputs, or lake systems could also become more nitrogen limited when for instance N‐fixing cyanobacteria blooms are favored by higher temperatures (Jones & Brett, 2014). Changes in species abundance ratios or even loss of species as a consequence of environmental changes can have detrimental knock‐on effects for ecosystem functioning. Such shifts can cause alterations in grazer populations and perpetuate further up the aquatic food chain. Other consequences may be that nutrients would be cycled differently, and carbon sequestered at varying rates (Henson et al., 2021). Here we show that species coexistence can be maintained when the thermal regime oscillates around the stable coexistence temperature. However, when thermal amplitudes become more extreme and deviate further from this temperature, changes in species abundances occur rapidly and the species displacement accelerates. Yet, other variables can interact and lead to unpredictable competition outcomes. We illustrate how nutrient limitation can diminish the competitiveness of the superior species and show that differences in a species' ability to recover from a thermal disturbance influence longer‐term community dynamics. If our findings apply to other phytoplankton functional groups, we suggest that aquatic heatwaves are likely to lead to abrupt and unpredictable restructuring of community composition.

## AUTHOR CONTRIBUTIONS

**Philipp Siegel:** Conceptualization (equal); data curation (lead); formal analysis (equal); investigation (lead); methodology (lead); project administration (lead); validation (lead); visualization (lead); writing – original draft (equal); writing – review and editing (equal). **Kirralee G. Baker:** Conceptualization (supporting); formal analysis (equal); supervision (supporting); visualization (supporting); funding acquisition (supporting); methodology (supporting); writing – original draft (equal); writing – review and editing (equal). **Etienne Low‐Décarie:** Conceptualization (supporting); formal analysis (supporting); funding acquisition (lead); methodology (supporting); resources (lead); supervision (equal); visualization (supporting); writing – original draft (supporting); writing – review and editing (supporting). **Richard Geider:** Conceptualization (supporting); formal analysis (supporting); funding acquisition (lead); methodology (supporting); supervision (equal); visualization (supporting); writing – original draft (supporting); writing – review and editing (supporting).

## CONFLICT OF INTEREST STATEMENT

The authors declare no competing interests.

## Supporting information


^**Appendix S1**^

## Data Availability

Collected data and R scripts used for the analysis can be downloaded via the publicly accessible repository “Knowledge Network for Biocomplexity” under the following link: https://knb.ecoinformatics.org/view/urn%3Auuid%3A4889f592‐096b‐48ee‐83c0‐c1e5b47a8bbc
