# Corrigendum

**DOI:** 10.1002/ece3.9982

**Published:** 2023-04-18

In the recent article by Siegel et al. (2023), the black dots in Figure 4 are missing. The correct figure is shown below.

**FIGURE 4:**
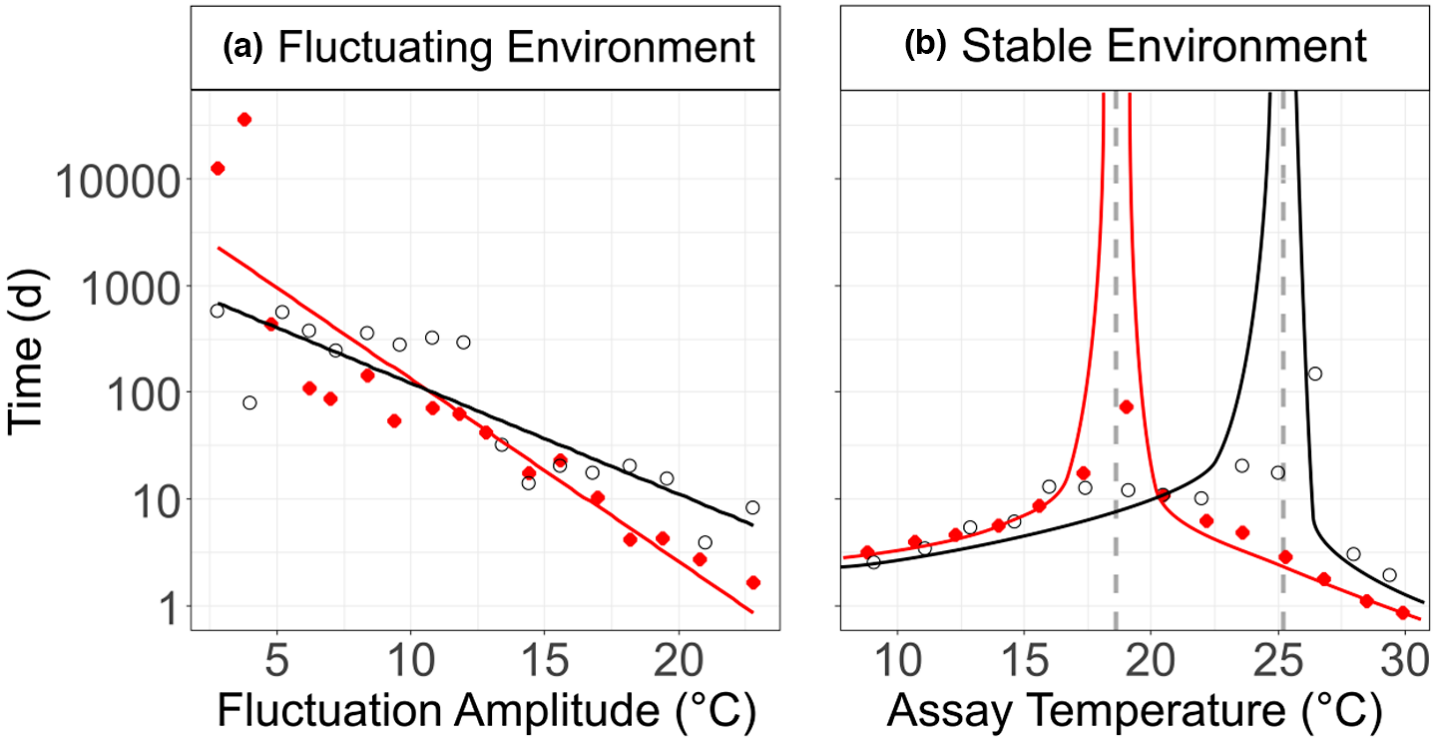
Time (days) taken to reach a 50% change in species abundance ratios in (a) fluctuating, and (b) stable temperature environments for high N (red) and low N (black) mixed cultures. Note that *y*‐axis is presented on a log10 scale to show that the time it takes encompasses periods from days and weeks to years. In (a), lines are from fitting linear models to the data. In (b), the lines are a visual aid to demonstrate that as the assay temperature approaches the temperature of stable coexistence, time approaches infinity. Gray dashed vertical lines visualize the temperature of stable coexistence in a stable thermal environment (18.6°C for high N and 25.2°C for low N mixed cultures).

The authors apologize for the error.
