# Supplementary material for: Phytoplankton competition and resilience under fluctuating temperature
Source: Ecol Evol. 2023 Mar 19;13(3):e9851. doi: 10.1002/ece3.9851 (PMC10025077; doi:10.1002/ece3.9851)
Supplement: Supplementary file 1 — Appendix S1 [file ECE3-13-e9851-s001.docx]

# Supplementary Material

## Supplementary Tables

Supplementary Table 1: Mixed cultures and the linear model parameter outputs fitted to the data of species abundance ratios over time in high N conditions before the start of the temperature fluctuations. Slopes significantly different from zero (P-value < 0.05) would be marked in bold and the corresponding row is coloured blue. P-values are Bonferroni-corrected to account for multiple testing of significance levels.

| **Replicate** | **Initial ratio of *P. tricornutum*-to-*T. pseudonana*** | **Direction of the Initial Fluctuation** | **Temperature Divergence from the Mean [°C]** | **Slope of Linear Regression [day^-1^]** | **R^2^ of Linear Regression** | **F-value** | **P-value** |
| --- | --- | --- | --- | --- | --- | --- | --- |
| 1 | 1 : 1 | down | 1.4 | 0.000704 | 0.003977 | 0.019963 | 1 |
| 1 | 1 : 1 | up | 1.4 | 0.005343 | 0.19093 | 0.943946 | 1 |
| 2 | 1 : 1 | down | 1.4 | -0.00062 | 0.006607 | 0.033254 | 1 |
| 2 | 1 : 1 | up | 1.4 | 0.000679 | 0.003545 | 0.014231 | 1 |
| 1 | 1 : 2 | down | 1.9 | -0.00521 | 0.136432 | 0.789933 | 1 |
| 1 | 1 : 2 | up | 1.9 | 0.002107 | 0.017038 | 0.069334 | 1 |
| 2 | 1 : 2 | down | 1.9 | 0.000847 | 0.008194 | 0.041306 | 1 |
| 2 | 1 : 2 | up | 1.9 | -0.00486 | 0.057256 | 0.242933 | 1 |
| 1 | 2 : 1 | down | 2.4 | -0.00276 | 0.07767 | 0.421051 | 1 |
| 1 | 2 : 1 | up | 2.4 | -0.00319 | 0.174994 | 0.848449 | 1 |
| 2 | 2 : 1 | down | 2.4 | -0.00152 | 0.017937 | 0.091325 | 1 |
| 2 | 2 : 1 | up | 2.4 | -0.00709 | 0.209862 | 1.062406 | 1 |
| 1 | 1 : 1 | down | 3.1 | -0.00497 | 0.134848 | 0.779333 | 1 |
| 1 | 1 : 1 | up | 3.1 | -0.00613 | 0.565896 | 5.214373 | 1 |
| 2 | 1 : 1 | down | 3.1 | -0.00361 | 0.087827 | 0.481415 | 1 |
| 2 | 1 : 1 | up | 3.1 | -0.0093 | 0.453396 | 4.147388 | 1 |
| 1 | 1 : 2 | down | 3.5 | -0.01953 | 0.54824 | 6.067828 | 1 |
| 1 | 1 : 2 | up | 3.5 | 0.001011 | 0.002046 | 0.0082 | 1 |
| 2 | 1 : 2 | down | 3.5 | 0.006169 | 0.076107 | 0.41188 | 1 |
| 2 | 1 : 2 | up | 3.5 | 0.001376 | 0.006727 | 0.027091 | 1 |
| 1 | 2 : 1 | down | 4.2 | -0.00144 | 0.057321 | 0.304033 | 1 |
| 1 | 2 : 1 | up | 4.2 | -0.00966 | 0.363671 | 2.286054 | 1 |
| 2 | 2 : 1 | down | 4.2 | -0.0013 | 0.023519 | 0.120425 | 1 |
| 2 | 2 : 1 | up | 4.2 | -0.001 | 0.012409 | 0.050258 | 1 |
| 1 | 1 : 1 | down | 4.7 | -0.00034 | 0.005485 | 0.027578 | 1 |
| 1 | 1 : 1 | up | 4.7 | 0.003825 | 0.134549 | 0.62187 | 1 |
| 2 | 1 : 1 | down | 4.7 | -0.0019 | 0.037747 | 0.235364 | 1 |
| 2 | 1 : 1 | up | 4.7 | 0.004823 | 0.314447 | 1.834708 | 1 |
| 1 | 1 : 2 | down | 5.4 | 0.000865 | 0.002664 | 0.013355 | 1 |
| 1 | 1 : 2 | up | 5.4 | -0.00804 | 0.193793 | 2.163388 | 1 |
| 2 | 1 : 2 | down | 5.4 | 0.003739 | 0.013177 | 0.066765 | 1 |
| 2 | 1 : 2 | up | 5.4 | 0.005877 | 0.259298 | 1.400283 | 1 |
| 1 | 2 : 1 | down | 5.9 | -0.00265 | 0.117873 | 0.66812 | 1 |
| 1 | 2 : 1 | up | 5.9 | 0.00265 | 0.057211 | 0.242732 | 1 |
| 2 | 2 : 1 | down | 5.9 | -0.00438 | 0.183422 | 1.123113 | 1 |
| 2 | 2 : 1 | up | 5.9 | -0.0012 | 0.013604 | 0.055167 | 1 |
| 1 | 1 : 1 | down | 6.4 | -0.00182 | 0.034764 | 0.180078 | 1 |
| 1 | 1 : 1 | up | 6.4 | 0.001868 | 0.033143 | 0.137118 | 1 |
| 2 | 1 : 1 | down | 6.4 | -0.00077 | 0.005859 | 0.029469 | 1 |
| 2 | 1 : 1 | up | 6.4 | -0.00069 | 0.002918 | 0.011707 | 1 |
| 1 | 1 : 2 | down | 7.2 | -0.00859 | 0.354031 | 4.932551 | 1 |
| 1 | 1 : 2 | up | 7.2 | -0.01549 | 0.388394 | 2.540156 | 1 |
| 2 | 1 : 2 | down | 7.2 | 0.008368 | 0.310502 | 2.251655 | 1 |
| 2 | 1 : 2 | up | 7.2 | 0.000257 | 9.54E-05 | 0.000382 | 1 |
| 1 | 2 : 1 | down | 7.8 | 0.001624 | 0.086482 | 0.473345 | 1 |
| 1 | 2 : 1 | up | 7.8 | -0.00153 | 0.057103 | 0.242243 | 1 |
| 2 | 2 : 1 | down | 7.8 | 0.003799 | 0.137919 | 0.799918 | 1 |
| 2 | 2 : 1 | up | 7.8 | -0.00018 | 0.001125 | 0.004507 | 1 |
| 1 | 1 : 1 | down | 8.5 | -0.00664 | 0.261092 | 1.766739 | 1 |
| 1 | 1 : 1 | up | 8.5 | -0.00745 | 0.447629 | 3.241511 | 1 |
| 2 | 1 : 1 | down | 8.5 | -0.00204 | 0.259887 | 1.755724 | 1 |
| 2 | 1 : 1 | up | 8.5 | -0.00941 | 0.538358 | 4.664724 | 1 |
| 1 | 1 : 2 | down | 9.1 | -0.00139 | 0.002743 | 0.013753 | 1 |
| 1 | 1 : 2 | up | 9.1 | 0.00125 | 0.004198 | 0.016861 | 1 |
| 2 | 1 : 2 | down | 9.1 | 0.010632 | 0.400026 | 3.3337 | 1 |
| 2 | 1 : 2 | up | 9.1 | -0.00426 | 0.058129 | 0.246864 | 1 |
| 1 | 2 : 1 | down | 9.7 | -0.00111 | 0.049251 | 0.259013 | 1 |
| 1 | 2 : 1 | up | 9.7 | -0.00177 | 0.019624 | 0.080069 | 1 |
| 2 | 2 : 1 | down | 9.7 | 0.006426 | 0.386626 | 3.151633 | 1 |
| 2 | 2 : 1 | up | 9.7 | -0.00205 | 0.030768 | 0.126978 | 1 |
| 1 | 1 : 2 | down | 10.4 | -0.00212 | 0.015861 | 0.080581 | 1 |
| 1 | 1 : 2 | up | 10.4 | -0.00512 | 0.115235 | 0.520976 | 1 |
| 2 | 1 : 2 | down | 10.4 | -0.00225 | 0.056808 | 0.301149 | 1 |
| 2 | 1 : 2 | up | 10.4 | 0.001742 | 0.006391 | 0.025727 | 1 |
| 1 | 1 : 1 | down | 11.4 | 0.002038 | 0.067682 | 0.362979 | 1 |
| 1 | 1 : 1 | up | 11.4 | -0.00312 | 0.152627 | 0.720472 | 1 |
| 2 | 1 : 1 | down | 11.4 | 0.000191 | 0.000739 | 0.003696 | 1 |
| 2 | 1 : 1 | up | 11.4 | -0.00225 | 0.019825 | 0.080902 | 1 |

Supplementary Table 2: Mixed cultures and the linear model parameter outputs fitted to the data of species abundance ratios over time in high N conditions during the temperature fluctuation period. Slopes significantly different from zero (P-value < 0.05) are marked in bold and the corresponding row is coloured blue. P-values are Bonferroni-corrected to account for multiple testing of significance levels.

| **Replicate** | **Initial ratio of *P. tricornutum*-to-*T. pseudonana*** | **Direction of the Initial Fluctuation** | **Temperature Divergence from the Mean [°C]** | **Slope of Linear Regression** | **R^2^ of Linear Regression [day^-1^]** | **F-value** | **P-value** |
| --- | --- | --- | --- | --- | --- | --- | --- |
| 1 | 1 : 1 | down | 1.4 | -0.00097 | 0.01682 | 0.325043 | 1 |
| 1 | 1 : 1 | up | 1.4 | 0.004026 | 0.198518 | 4.706077 | 1 |
| 2 | 1 : 1 | down | 1.4 | 0.000437 | 0.005138 | 0.098118 | 1 |
| 2 | 1 : 1 | up | 1.4 | -0.00327 | 0.166741 | 3.802046 | 1 |
| 1 | 1 : 2 | down | 1.9 | -0.00168 | 0.028932 | 0.208561 | 1 |
| 1 | 1 : 2 | up | 1.9 | 0.013813 | 0.545621 | 8.405658 | 1 |
| 2 | 1 : 2 | down | 1.9 | 0.000923 | 0.006651 | 0.04687 | 1 |
| 2 | 1 : 2 | up | 1.9 | -0.01298 | 0.380314 | 4.296036 | 1 |
| 1 | 2 : 1 | down | 2.4 | -0.00843 | 0.561595 | 8.966987 | 1 |
| 1 | 2 : 1 | up | 2.4 | 0.000324 | 0.005079 | 0.035733 | 1 |
| 2 | 2 : 1 | down | 2.4 | 0.002583 | 0.134027 | 1.083393 | 1 |
| 2 | 2 : 1 | up | 2.4 | -0.00095 | 0.016004 | 0.113849 | 1 |
| 1 | 1 : 1 | down | 3.1 | -0.00507 | 0.676094 | 14.61123 | 0.443428 |
| 1 | 1 : 1 | up | 3.1 | -0.00183 | 0.051827 | 0.382618 | 1 |
| 2 | 1 : 1 | down | 3.1 | -0.00945 | 0.787642 | 25.96326 | 0.095676 |
| 2 | 1 : 1 | up | 3.1 | -0.00881 | 0.80908 | 29.66455 | 0.06528 |
| 1 | 1 : 2 | down | 3.5 | -0.03245 | 0.77575 | 24.21521 | 0.116416 |
| 1 | 1 : 2 | up | 3.5 | 0.014117 | 0.408441 | 4.833147 | 1 |
| 2 | 1 : 2 | down | 3.5 | -0.00461 | 0.320969 | 3.308804 | 1 |
| 2 | 1 : 2 | up | 3.5 | -0.00903 | 0.260083 | 2.460515 | 1 |
| 1 | 2 : 1 | down | 4.2 | -0.00532 | 0.374997 | 4.19995 | 1 |
| 1 | 2 : 1 | up | 4.2 | 0.002341 | 0.081515 | 0.621249 | 1 |
| 2 | 2 : 1 | down | 4.2 | -0.00197 | 0.098504 | 0.764875 | 1 |
| 2 | 2 : 1 | up | 4.2 | -0.01432 | 0.767289 | 23.08022 | 0.133076 |
| 1 | 1 : 1 | down | 4.7 | -0.01472 | 0.654995 | 13.28958 | 0.559436 |
| 1 | 1 : 1 | up | 4.7 | -0.0032 | 0.119827 | 0.952982 | 1 |
| 2 | 1 : 1 | down | 4.7 | -0.01367 | 0.785258 | 25.59729 | 0.099552 |
| 2 | 1 : 1 | up | 4.7 | -0.01956 | 0.793077 | 26.82901 | 0.087108 |
| 1 | 1 : 2 | down | 5.4 | -0.01542 | 0.396382 | 4.596745 | 1 |
| 1 | 1 : 2 | up | 5.4 | 0.000666 | 0.0025 | 0.017544 | 1 |
| 2 | 1 : 2 | down | 5.4 | -0.00699 | 0.136653 | 1.107981 | 1 |
| 2 | 1 : 2 | up | 5.4 | -0.01748 | 0.671019 | 14.27784 | 0.46954 |
| 1 | 2 : 1 | down | 5.9 | -0.01131 | 0.589269 | 10.04277 | 1 |
| 1 | 2 : 1 | up | 5.9 | -0.00124 | 0.033697 | 0.244106 | 1 |
| 2 | 2 : 1 | down | 5.9 | -0.00892 | 0.528024 | 7.831262 | 1 |
| 2 | 2 : 1 | up | 5.9 | -0.02314 | 0.946853 | 124.7109 | **0.0007** |
| 1 | 1 : 1 | down | 6.4 | -0.01765 | 0.854133 | 40.98902 | **0.024888** |
| 1 | 1 : 1 | up | 6.4 | -0.01448 | 0.584582 | 9.850488 | 1 |
| 2 | 1 : 1 | down | 6.4 | -0.01606 | 0.661671 | 13.68992 | 0.52054 |
| 2 | 1 : 1 | up | 6.4 | -0.01842 | 0.776256 | 24.28581 | 0.115464 |
| 1 | 1 : 2 | down | 7.2 | -0.06065 | 0.753582 | 21.40698 | 0.163676 |
| 1 | 1 : 2 | up | 7.2 | -0.01837 | 0.210235 | 1.863396 | 1 |
| 2 | 1 : 2 | down | 7.2 | -0.04853 | 0.840791 | 36.96747 | **0.034068** |
| 2 | 1 : 2 | up | 7.2 | -0.03158 | 0.430578 | 5.293166 | 1 |
| 1 | 2 : 1 | down | 7.8 | -0.02579 | 0.902995 | 65.16152 | **0.005855** |
| 1 | 2 : 1 | up | 7.8 | -0.02223 | 0.831078 | 34.43916 | **0.042092** |
| 2 | 2 : 1 | down | 7.8 | -0.01796 | 0.852024 | 40.30498 | **0.026248** |
| 2 | 2 : 1 | up | 7.8 | -0.05367 | 0.912444 | 72.94926 | **0.004073** |
| 1 | 1 : 1 | down | 8.5 | -0.06398 | 0.84717 | 72.0618 | **7.89E-05** |
| 1 | 1 : 1 | up | 8.5 | -0.05116 | 0.822492 | 60.23609 | **0.000211** |
| 2 | 1 : 1 | down | 8.5 | -0.04449 | 0.838821 | 67.65581 | **0.000112** |
| 2 | 1 : 1 | up | 8.5 | -0.10837 | 0.855077 | 76.70278 | **5.57E-05** |
| 1 | 1 : 2 | down | 9.1 | -0.17695 | 0.857676 | 42.18358 | **0.022848** |
| 1 | 1 : 2 | up | 9.1 | -0.1382 | 0.841608 | 37.19406 | **0.033456** |
| 2 | 1 : 2 | down | 9.1 | -0.15406 | 0.820594 | 32.01761 | **0.052224** |
| 2 | 1 : 2 | up | 9.1 | -0.19552 | 0.894427 | 50.83289 | **0.026044** |
| 1 | 2 : 1 | down | 9.7 | -0.17383 | 0.858497 | 42.46888 | **0.022372** |
| 1 | 2 : 1 | up | 9.7 | -0.09608 | 0.89397 | 59.0188 | **0.008024** |
| 2 | 2 : 1 | down | 9.7 | -0.17471 | 0.758567 | 21.99353 | **0.15198** |
| 2 | 2 : 1 | up | 9.7 | -0.20408 | 0.895933 | 60.2641 | **0.00748** |
| 1 | 1 : 2 | down | 10.4 | -0.22104 | 0.956519 | 109.9933 | **0.009248** |
| 1 | 1 : 2 | up | 10.4 | -0.29297 | 0.927651 | 76.93177 | **0.008296** |
| 2 | 1 : 2 | down | 10.4 | -0.27592 | 0.913072 | 52.51864 | **0.053108** |
| 2 | 1 : 2 | up | 10.4 | -0.24031 | 0.895776 | 42.97349 | **0.084184** |
| 1 | 1 : 1 | down | 11.4 | -0.41616 | 0.921465 | 117.3324 | **5.17E-05** |
| 1 | 1 : 1 | up | 11.4 | -0.4683 | 0.948239 | 183.1941 | **6.36E-06** |
| 2 | 1 : 1 | down | 11.4 | -0.39704 | 0.925922 | 137.4916 | **1E-05** |
| 2 | 1 : 1 | up | 11.4 | -0.41986 | 0.927006 | 126.9979 | **3.58E-05** |

Supplementary Table 3: Mixed cultures and the linear model parameter outputs fitted to the data of species abundance ratios over time in high N conditions after the temperature fluctuations. Slopes significantly different from zero (P-value < 0.05) are marked in bold and the corresponding row is coloured blue. P-values are Bonferroni-corrected to account for multiple testing of significance levels.

| **Replicate** | **Initial ratio of *P. tricornutum*-to-*T. pseudonana*** | **Direction of the Initial Fluctuation** | **Temperature Divergence from the Mean [°C]** | **Slope of Linear Regression [day^-1^]** | **R^2^ of Linear Regression** | **F-value** | **P-value** |
| --- | --- | --- | --- | --- | --- | --- | --- |
| 1 | 1 : 1 | down | 1.4 | -0.0041 | 0.462099 | 2.577231 | 1 |
| 1 | 1 : 1 | up | 1.4 | 0.002704 | 0.031097 | 0.128382 | 1 |
| 2 | 1 : 1 | down | 1.4 | 0.001347 | 0.028172 | 0.086967 | 1 |
| 2 | 1 : 1 | up | 1.4 | 0.007341 | 0.627475 | 6.737535 | 1 |
| 1 | 1 : 2 | down | 1.9 | 0.001884 | 0.007979 | 0.024128 | 1 |
| 1 | 1 : 2 | up | 1.9 | -0.01274 | 0.406237 | 2.736695 | 1 |
| 2 | 1 : 2 | down | 1.9 | -0.00018 | 2.26E-05 | 6.77E-05 | 1 |
| 2 | 1 : 2 | up | 1.9 | 0.005311 | 0.046637 | 0.195675 | 1 |
| 1 | 2 : 1 | down | 2.4 | -0.00013 | 9.24E-05 | 0.000277 | 1 |
| 1 | 2 : 1 | up | 2.4 | 0.003283 | 0.138179 | 0.641333 | 1 |
| 2 | 2 : 1 | down | 2.4 | 0.003087 | 0.303817 | 1.309215 | 1 |
| 2 | 2 : 1 | up | 2.4 | 0.000857 | 0.005961 | 0.023987 | 1 |
| 1 | 1 : 1 | down | 3.1 | 0.004765 | 0.234399 | 0.918493 | 1 |
| 1 | 1 : 1 | up | 3.1 | 0.006752 | 0.357544 | 2.226104 | 1 |
| 2 | 1 : 1 | down | 3.1 | 0.003201 | 0.110833 | 0.373945 | 1 |
| 2 | 1 : 1 | up | 3.1 | -0.00097 | 0.00248 | 0.009944 | 1 |
| 1 | 1 : 2 | down | 3.5 | 0.001562 | 0.005864 | 0.017695 | 1 |
| 1 | 1 : 2 | up | 3.5 | 0.015871 | 0.341045 | 2.070215 | 1 |
| 2 | 1 : 2 | down | 3.5 | -0.00514 | 0.015227 | 0.046387 | 1 |
| 2 | 1 : 2 | up | 3.5 | -0.00333 | 0.028214 | 0.116131 | 1 |
| 1 | 2 : 1 | down | 4.2 | -0.00182 | 0.084655 | 0.277454 | 1 |
| 1 | 2 : 1 | up | 4.2 | -0.00358 | 0.636541 | 7.005355 | 1 |
| 2 | 2 : 1 | down | 4.2 | -0.00071 | 0.007272 | 0.021975 | 1 |
| 2 | 2 : 1 | up | 4.2 | 0.000787 | 0.035348 | 0.146574 | 1 |
| 1 | 1 : 1 | down | 4.7 | 0.025776 | 0.809194 | 12.72278 | 1 |
| 1 | 1 : 1 | up | 4.7 | 0.006883 | 0.153356 | 0.724537 | 1 |
| 2 | 1 : 1 | down | 4.7 | 0.035379 | 0.94027 | 47.22568 | 0.3786 |
| 2 | 1 : 1 | up | 4.7 | 0.021125 | 0.465278 | 3.480524 | 1 |
| 1 | 1 : 2 | down | 5.4 | 0.026477 | 0.600438 | 4.508229 | 1 |
| 1 | 1 : 2 | up | 5.4 | 0.002357 | 0.006252 | 0.025163 | 1 |
| 2 | 1 : 2 | down | 5.4 | 0.037116 | 0.602204 | 4.541547 | 1 |
| 2 | 1 : 2 | up | 5.4 | -2.8E-05 | 3.56E-07 | 1.42E-06 | 1 |
| 1 | 2 : 1 | down | 5.9 | 0.006679 | 0.121503 | 0.414922 | 1 |
| 1 | 2 : 1 | up | 5.9 | 0.006887 | 0.341655 | 2.075844 | 1 |
| 2 | 2 : 1 | down | 5.9 | -0.00018 | 0.000211 | 0.000634 | 1 |
| 2 | 2 : 1 | up | 5.9 | 0.0036 | 0.277276 | 1.534616 | 1 |
| 1 | 1 : 1 | down | 6.4 | 0.028175 | 0.994489 | 541.3466 | **0.01044** |
| 1 | 1 : 1 | up | 6.4 | 0.018822 | 0.536964 | 4.638633 | 1 |
| 2 | 1 : 1 | down | 6.4 | 0.011348 | 0.198177 | 0.741474 | 1 |
| 2 | 1 : 1 | up | 6.4 | 0.011588 | 0.578283 | 5.485032 | 1 |
| 1 | 1 : 2 | down | 7.2 | 0.013724 | 0.100282 | 0.334379 | 1 |
| 1 | 1 : 2 | up | 7.2 | 0.036911 | 0.146181 | 0.684832 | 1 |
| 2 | 1 : 2 | down | 7.2 | 0.009347 | 0.03627 | 0.112905 | 1 |
| 2 | 1 : 2 | up | 7.2 | 0.009962 | 0.09416 | 0.415792 | 1 |
| 1 | 2 : 1 | down | 7.8 | 0.011405 | 0.316249 | 1.387561 | 1 |
| 1 | 2 : 1 | up | 7.8 | 0.004467 | 0.072219 | 0.311362 | 1 |
| 2 | 2 : 1 | down | 7.8 | 0.005259 | 0.079561 | 0.259316 | 1 |
| 2 | 2 : 1 | up | 7.8 | 0.020324 | 0.39413 | 2.602074 | 1 |
| 1 | 1 : 1 | down | 8.5 | 0.053806 | 0.182997 | 0.671956 | 1 |
| 1 | 1 : 1 | up | 8.5 | -0.01352 | 0.086257 | 0.377597 | 1 |
| 2 | 1 : 1 | down | 8.5 | 0.022243 | 0.907026 | 29.26711 | 0.7431 |
| 2 | 1 : 1 | up | 8.5 | 0.012035 | 0.013937 | 0.056535 | 1 |
| 1 | 1 : 2 | down | 9.1 | 0.00212 | 0.000221 | 0.000662 | 1 |
| 1 | 1 : 2 | up | 9.1 | 0.053457 | 0.368693 | 2.33606 | 1 |
| 2 | 1 : 2 | down | 9.1 | -0.04168 | 0.120945 | 0.412756 | 1 |
| 2 | 1 : 2 | up | 9.1 | 2.76E-16 | 0 | 0 | 1 |
| 1 | 2 : 1 | down | 9.7 | 0.084815 | 0.150835 | 0.532881 | 1 |
| 1 | 2 : 1 | up | 9.7 | 0.088437 | 0.74929 | 11.95466 | 1 |
| 2 | 2 : 1 | down | 9.7 | 0.067986 | 0.836151 | 15.30951 | 1 |
| 2 | 2 : 1 | up | 9.7 | 0.179629 | 0.442194 | 2.378213 | 1 |

Supplementary Table 4: Mixed cultures and the linear model parameter outputs fitted to the data of species abundance ratios over time in low N conditions before the start of the temperature fluctuations. Slopes significantly different from zero (P-value < 0.05) are marked in bold and the corresponding row is coloured blue. P-values are Bonferroni-corrected to account for multiple testing of significance levels.

| **Replicate** | **Initial ratio of *P. tricornutum*-to-*T. pseudonana*** | **Direction of the Initial Fluctuation** | **Temperature Divergence from the Mean [°C]** | **Slope of Linear Regression [day^-1^]** | **R^2^ of Linear Regression** | **F-value** | **P-value** |
| --- | --- | --- | --- | --- | --- | --- | --- |
| 1 | 1 : 1 | down | 1.4 | -0.00207 | 0.022511 | 0.115147 | 1 |
| 1 | 1 : 1 | up | 1.4 | -0.00295 | 0.064131 | 0.274102 | 1 |
| 2 | 1 : 1 | down | 1.4 | -0.00429 | 0.092991 | 0.512622 | 1 |
| 2 | 1 : 1 | up | 1.4 | -0.00601 | 0.117152 | 0.530791 | 1 |
| 1 | 1 : 2 | down | 2 | -0.01259 | 0.588811 | 7.159871 | 1 |
| 1 | 1 : 2 | up | 2 | 0.001154 | 0.003698 | 0.014846 | 1 |
| 2 | 1 : 2 | down | 2 | -0.00502 | 0.121094 | 0.688891 | 1 |
| 2 | 1 : 2 | up | 2 | -0.00308 | 0.017582 | 0.071586 | 1 |
| 1 | 2 : 1 | down | 2.6 | 0.000653 | 0.007393 | 0.037239 | 1 |
| 1 | 2 : 1 | up | 2.6 | -0.00548 | 0.218972 | 1.121457 | 1 |
| 2 | 2 : 1 | down | 2.6 | 0.00511 | 0.19379 | 1.201862 | 1 |
| 2 | 2 : 1 | up | 2.6 | 0.004745 | 0.458032 | 3.380505 | 1 |
| 1 | 1 : 1 | down | 3.1 | 0.000495 | 0.001902 | 0.00953 | 1 |
| 1 | 1 : 1 | up | 3.1 | -0.00011 | 0.000256 | 0.001023 | 1 |
| 2 | 1 : 1 | down | 3.1 | -0.00416 | 0.199122 | 1.24315 | 1 |
| 2 | 1 : 1 | up | 3.1 | 0.001145 | 0.008801 | 0.035517 | 1 |
| 1 | 1 : 2 | down | 3.6 | 0.008292 | 0.389812 | 3.194203 | 1 |
| 1 | 1 : 2 | up | 3.6 | 0.015461 | 0.52417 | 4.406355 | 1 |
| 2 | 1 : 2 | down | 3.6 | -0.01137 | 0.204388 | 1.284473 | 1 |
| 2 | 1 : 2 | up | 3.6 | -0.01041 | 0.346092 | 2.11707 | 1 |
| 1 | 2 : 1 | down | 4.2 | 0.003308 | 0.274755 | 1.894222 | 1 |
| 1 | 2 : 1 | up | 4.2 | -0.00275 | 0.080583 | 0.350583 | 1 |
| 2 | 2 : 1 | down | 4.2 | -0.00217 | 0.026078 | 0.133881 | 1 |
| 2 | 2 : 1 | up | 4.2 | 0.003194 | 0.153914 | 0.727654 | 1 |
| 1 | 1 : 1 | down | 4.8 | -0.00354 | 0.200943 | 1.257375 | 1 |
| 1 | 1 : 1 | up | 4.8 | -0.00087 | 0.018722 | 0.076318 | 1 |
| 2 | 1 : 1 | down | 4.8 | -0.00537 | 0.233117 | 1.519902 | 1 |
| 2 | 1 : 1 | up | 4.8 | 0.000601 | 0.002632 | 0.010554 | 1 |
| 1 | 1 : 2 | down | 5.4 | 0.009272 | 0.192597 | 1.192696 | 1 |
| 1 | 1 : 2 | up | 5.4 | 0.005692 | 0.041236 | 0.17204 | 1 |
| 2 | 1 : 2 | down | 5.4 | 0.000171 | 0.000115 | 0.000574 | 1 |
| 2 | 1 : 2 | up | 5.4 | 0.007954 | 0.28459 | 1.591197 | 1 |
| 1 | 2 : 1 | down | 6 | -0.00048 | 0.023658 | 0.121156 | 1 |
| 1 | 2 : 1 | up | 6 | 0.006886 | 0.655813 | 7.621584 | 1 |
| 2 | 2 : 1 | down | 6 | 0.005404 | 0.419454 | 3.612583 | 1 |
| 2 | 2 : 1 | up | 6 | 0.004647 | 0.189516 | 0.935325 | 1 |
| 1 | 1 : 1 | down | 6.7 | -0.00233 | 0.047289 | 0.24818 | 1 |
| 1 | 1 : 1 | up | 6.7 | 0.002156 | 0.040527 | 0.168956 | 1 |
| 2 | 1 : 1 | down | 6.7 | -0.00305 | 0.108801 | 0.610417 | 1 |
| 2 | 1 : 1 | up | 6.7 | 0.003112 | 0.111682 | 0.502894 | 1 |
| 1 | 1 : 2 | down | 7.2 | 0.01508 | 0.415639 | 3.556352 | 1 |
| 1 | 1 : 2 | up | 7.2 | 0.006433 | 0.261302 | 1.414932 | 1 |
| 2 | 1 : 2 | down | 7.2 | 0.014497 | 0.282026 | 1.964042 | 1 |
| 2 | 1 : 2 | up | 7.2 | 0.014787 | 0.484179 | 3.754625 | 1 |
| 1 | 2 : 1 | down | 7.8 | -0.00464 | 0.37905 | 3.052179 | 1 |
| 1 | 2 : 1 | up | 7.8 | 0.003467 | 0.164953 | 0.790147 | 1 |
| 2 | 2 : 1 | down | 7.8 | 0.001968 | 0.121156 | 0.689289 | 1 |
| 2 | 2 : 1 | up | 7.8 | -0.00034 | 0.002359 | 0.009459 | 1 |
| 1 | 1 : 1 | down | 8.4 | -0.00641 | 0.363611 | 2.856831 | 1 |
| 1 | 1 : 1 | up | 8.4 | -0.00264 | 0.147206 | 0.690464 | 1 |
| 2 | 1 : 1 | down | 8.4 | -0.00352 | 0.097469 | 0.539976 | 1 |
| 2 | 1 : 1 | up | 8.4 | 0.000474 | 0.001408 | 0.005642 | 1 |
| 1 | 1 : 2 | down | 9.1 | 0.010991 | 0.263912 | 1.792664 | 1 |
| 1 | 1 : 2 | up | 9.1 | 0.001973 | 0.017564 | 0.071512 | 1 |
| 2 | 1 : 2 | down | 9.1 | 0.000751 | 0.004253 | 0.021358 | 1 |
| 2 | 1 : 2 | up | 9.1 | 0.023603 | 0.506124 | 4.099196 | 1 |
| 1 | 2 : 1 | down | 9.8 | -0.00214 | 0.086903 | 0.475868 | 1 |
| 1 | 2 : 1 | up | 9.8 | 0.004627 | 0.264647 | 1.439563 | 1 |
| 2 | 2 : 1 | down | 9.8 | 0.005225 | 0.406606 | 3.426112 | 1 |
| 2 | 2 : 1 | up | 9.8 | 0.004776 | 0.354596 | 2.197665 | 1 |
| 1 | 1 : 2 | down | 10.5 | -0.00299 | 0.045246 | 0.236953 | 1 |
| 1 | 1 : 2 | up | 10.5 | -0.0098 | 0.269353 | 1.474596 | 1 |
| 2 | 1 : 2 | down | 10.5 | 0.00258 | 0.010738 | 0.054273 | 1 |
| 2 | 1 : 2 | up | 10.5 | 0.011702 | 0.317343 | 1.859457 | 1 |
| 1 | 1 : 1 | down | 11.4 | 0.004794 | 0.133934 | 0.773231 | 1 |
| 1 | 1 : 1 | up | 11.4 | -0.00172 | 0.025442 | 0.104424 | 1 |
| 2 | 1 : 1 | down | 11.4 | 0.001799 | 0.050421 | 0.265493 | 1 |
| 2 | 1 : 1 | up | 11.4 | -0.00186 | 0.041565 | 0.173469 | 1 |

Supplementary Table 5: Mixed cultures and the linear model parameter outputs fitted to the data of species abundance ratios over time in low N conditions during the temperature fluctuation period. Slopes significantly different from zero (P-value < 0.05) are marked in bold and the corresponding row is coloured blue. P-values are Bonferroni-corrected to account for multiple testing of significance levels. Rows with fluctuation amplitudes for which *P. tricornutum* cells were fully dead within one “warm” fluctuation cycle are marked in pink (grey-shaded area in main text figure 3B); the linear models were fit on available data that has very large manual counting error and the results have no biological relevance.

| **Replicate** | **Initial ratio of *P. tricornutum*-to-*T. pseudonana*** | **Direction of the Initial Fluctuation** | **Temperature Divergence from the Mean [°C]** | **Slope of Linear Regression [day^-1^]** | **R^2^ of Linear Regression** | **F-value** | **P-value** |
| --- | --- | --- | --- | --- | --- | --- | --- |
| 1 | 1 : 1 | down | 1.4 | 0.006803 | 0.388899 | 7.000309 | 1 |
| 1 | 1 : 1 | up | 1.4 | 0.007418 | 0.507287 | 11.32537 | 0.428672 |
| 2 | 1 : 1 | down | 1.4 | 0.005363 | 0.643284 | 19.83685 | 0.066096 |
| 2 | 1 : 1 | up | 1.4 | 0.004811 | 0.517875 | 11.81568 | 0.3774 |
| 1 | 1 : 2 | down | 2 | 0.00925 | 0.129063 | 1.630074 | 1 |
| 1 | 1 : 2 | up | 2 | -0.00287 | 0.01511 | 0.168763 | 1 |
| 2 | 1 : 2 | down | 2 | 0.015589 | 0.412619 | 7.727194 | 1 |
| 2 | 1 : 2 | up | 2 | -0.00151 | 0.003615 | 0.039907 | 1 |
| 1 | 2 : 1 | down | 2.6 | 0.004704 | 0.274616 | 4.164388 | 1 |
| 1 | 2 : 1 | up | 2.6 | 0.00571 | 0.329931 | 5.416212 | 1 |
| 2 | 2 : 1 | down | 2.6 | 0.003258 | 0.354344 | 6.036927 | 1 |
| 2 | 2 : 1 | up | 2.6 | 0.003061 | 0.124839 | 1.569121 | 1 |
| 1 | 1 : 1 | down | 3.1 | 0.008249 | 0.712413 | 27.24932 | **0.01938** |
| 1 | 1 : 1 | up | 3.1 | 0.011138 | 0.793886 | 42.36857 | **0.002972** |
| 2 | 1 : 1 | down | 3.1 | 0.0071 | 0.542896 | 13.06455 | 0.27642 |
| 2 | 1 : 1 | up | 3.1 | 0.007253 | 0.66632 | 21.9657 | **0.045152** |
| 1 | 1 : 2 | down | 3.6 | 0.004377 | 0.039577 | 0.45329 | 1 |
| 1 | 1 : 2 | up | 3.6 | 0.002062 | 0.005561 | 0.061517 | 1 |
| 2 | 1 : 2 | down | 3.6 | 0.005344 | 0.098753 | 1.205313 | 1 |
| 2 | 1 : 2 | up | 3.6 | -0.00106 | 0.004123 | 0.045538 | 1 |
| 1 | 2 : 1 | down | 4.2 | -0.00169 | 0.08647 | 0.946545 | 1 |
| 1 | 2 : 1 | up | 4.2 | -0.00046 | 0.003318 | 0.036616 | 1 |
| 2 | 2 : 1 | down | 4.2 | -0.00481 | 0.430465 | 7.55818 | 1 |
| 2 | 2 : 1 | up | 4.2 | -0.00367 | 0.279816 | 3.885333 | 1 |
| 1 | 1 : 1 | down | 4.8 | -0.00813 | 0.713065 | 27.33621 | **0.019176** |
| 1 | 1 : 1 | up | 4.8 | -0.00796 | 0.570586 | 14.61629 | 0.192304 |
| 2 | 1 : 1 | down | 4.8 | -0.01082 | 0.619193 | 17.88601 | 0.096152 |
| 2 | 1 : 1 | up | 4.8 | -0.01319 | 0.71494 | 27.58843 | **0.018496** |
| 1 | 1 : 2 | down | 5.4 | -0.03746 | 0.741059 | 31.48071 | **0.010744** |
| 1 | 1 : 2 | up | 5.4 | -0.03351 | 0.726805 | 29.26425 | **0.014552** |
| 2 | 1 : 2 | down | 5.4 | -0.03835 | 0.790325 | 41.46217 | **0.003271** |
| 2 | 1 : 2 | up | 5.4 | -0.03526 | 0.72186 | 28.54843 | **0.016048** |
| 1 | 2 : 1 | down | 6 | -0.04552 | 0.960807 | 245.1485 | **1.57E-06** |
| 1 | 2 : 1 | up | 6 | -0.04123 | 0.969847 | 321.6417 | **4.22E-07** |
| 2 | 2 : 1 | down | 6 | -0.04591 | 0.970687 | 331.1464 | **3.67E-07** |
| 2 | 2 : 1 | up | 6 | -0.04191 | 0.894797 | 85.05412 | **0.000226** |
| 1 | 1 : 1 | down | 6.7 | -0.10954 | 0.940076 | 172.5674 | **3.11E-06** |
| 1 | 1 : 1 | up | 6.7 | -0.08846 | 0.988814 | 972.4115 | **2.98E-10** |
| 2 | 1 : 1 | down | 6.7 | -0.14062 | 0.946865 | 196.0211 | **1.6E-06** |
| 2 | 1 : 1 | up | 6.7 | -0.10638 | 0.979486 | 525.2122 | **8.36E-09** |
| 1 | 1 : 2 | down | 7.2 | -0.20236 | 0.915641 | 86.83269 | **0.000972** |
| 1 | 1 : 2 | up | 7.2 | -0.20316 | 0.982499 | 449.1283 | **1.75E-06** |
| 2 | 1 : 2 | down | 7.2 | -0.2968 | 0.919595 | 91.4969 | **0.000802** |
| 2 | 1 : 2 | up | 7.2 | -0.18808 | 0.971624 | 273.9331 | **1.22E-05** |
| 1 | 2 : 1 | down | 7.8 | -0.18385 | 0.892506 | 24.90849 | 1 |
| 1 | 2 : 1 | up | 7.8 | -0.25359 | 0.958457 | 92.28597 | **0.044608** |
| 2 | 2 : 1 | down | 7.8 | -0.2557 | 0.837163 | 15.42332 | 1 |
| 2 | 2 : 1 | up | 7.8 | -0.24411 | 0.936766 | 59.25682 | 0.104176 |
| 1 | 1 : 1 | down | 8.4 | -0.48317 | 0.820629 | 13.72511 | 1 |
| 1 | 1 : 1 | up | 8.4 | -0.5693 | 0.740708 | 11.42662 | 1 |
| 2 | 1 : 1 | down | 8.4 | -0.49645 | 0.870929 | 20.24308 | 1 |
| 2 | 1 : 1 | up | 8.4 | -0.4905 | 0.781696 | 14.32311 | 1 |
| 1 | 1 : 2 | down | 9.1 | -0.29692 | 0.95625 | 65.57082 | 0.267648 |
| 1 | 1 : 2 | up | 9.1 | -0.34638 | 0.945482 | 69.3696 | 0.077248 |
| 2 | 1 : 2 | down | 9.1 | -0.30742 | 0.959342 | 70.7867 | 0.239564 |
| 2 | 1 : 2 | up | 9.1 | -0.36988 | 0.967731 | 119.9592 | **0.02686** |
| 1 | 2 : 1 | down | 9.8 | -0.23024 | 0.747184 | 5.910884 | 1 |
| 1 | 2 : 1 | up | 9.8 | -0.30603 | 0.896385 | 25.95341 | 0.994296 |
| 2 | 2 : 1 | down | 9.8 | -0.23871 | 0.828684 | 9.67436 | 1 |
| 2 | 2 : 1 | up | 9.8 | -0.3179 | 0.910375 | 30.47292 | 0.796212 |
| 1 | 1 : 2 | down | 10.5 | -0.36904 | 0.936656 | 29.57387 | 1 |
| 1 | 1 : 2 | up | 10.5 | -0.67626 | 0.80513 | 12.39485 | 1 |
| 2 | 1 : 2 | down | 10.5 | -0.31617 | 0.929921 | 26.53942 | 1 |
| 2 | 1 : 2 | up | 10.5 | -0.63056 | 0.840013 | 15.75156 | 1 |
| 1 | 1 : 1 | down | 11.4 | -0.27432 | 0.538309 | 1.16595 | 1 |
| 1 | 1 : 1 | up | 11.4 | -0.45734 | 0.670797 | 4.075285 | 1 |
| 2 | 1 : 1 | down | 11.4 | -0.31538 | 0.508826 | 1.035939 | 1 |
| 2 | 1 : 1 | up | 11.4 | -0.41034 | 0.68472 | 4.343561 | 1 |

Supplementary Table 6: Mixed cultures and the linear model parameter outputs fitted to the data of species abundance ratios over time in low N conditions after the temperature fluctuations. Slopes significantly different from zero (P-value < 0.05) are marked in bold and the corresponding row is coloured blue. P-values are Bonferroni-corrected to account for multiple testing of significance levels.

| **Replicate** | **Initial ratio of *P. tricornutum*-to-*T. pseudonana*** | **Direction of the Initial Fluctuation** | **Temperature Divergence from the Mean [°C]** | **Slope of Linear Regression [day^-1^]** | **R^2^ of Linear Regression** | **F-value** | **P-value** |
| --- | --- | --- | --- | --- | --- | --- | --- |
| 1 | 1 : 1 | down | 1.4 | 0.009181 | 0.441353 | 2.370116 | 1 |
| 1 | 1 : 1 | up | 1.4 | 0.01028 | 0.512054 | 4.197626 | 1 |
| 2 | 1 : 1 | down | 1.4 | 0.002886 | 0.260634 | 1.057528 | 1 |
| 2 | 1 : 1 | up | 1.4 | 0.010635 | 0.705524 | 9.58346 | 1 |
| 1 | 1 : 2 | down | 2 | 0.028298 | 0.490043 | 2.882852 | 1 |
| 1 | 1 : 2 | up | 2 | 0.013899 | 0.264204 | 1.436287 | 1 |
| 2 | 1 : 2 | down | 2 | 0.017923 | 0.626519 | 5.032541 | 1 |
| 2 | 1 : 2 | up | 2 | 0.010346 | 0.34207 | 2.079671 | 1 |
| 1 | 2 : 1 | down | 2.6 | 0.007378 | 0.698619 | 6.954182 | 1 |
| 1 | 2 : 1 | up | 2.6 | 0.009994 | 0.586716 | 5.678568 | 1 |
| 2 | 2 : 1 | down | 2.6 | 0.00569 | 0.330071 | 1.478087 | 1 |
| 2 | 2 : 1 | up | 2.6 | -0.0031 | 0.306657 | 1.769147 | 1 |
| 1 | 1 : 1 | down | 3.1 | 0.012668 | 0.864032 | 19.06405 | 0.955288 |
| 1 | 1 : 1 | up | 3.1 | 0.007242 | 0.492853 | 3.887264 | 1 |
| 2 | 1 : 1 | down | 3.1 | 0.005862 | 0.874199 | 20.84718 | 0.847272 |
| 2 | 1 : 1 | up | 3.1 | 0.01369 | 0.854012 | 23.39942 | 0.361931 |
| 1 | 1 : 2 | down | 3.6 | 0.038285 | 0.937557 | 45.04398 | 0.290293 |
| 1 | 1 : 2 | up | 3.6 | 0.02467 | 0.722338 | 10.40599 | 1 |
| 2 | 1 : 2 | down | 3.6 | 0.017014 | 0.427534 | 2.240483 | 1 |
| 2 | 1 : 2 | up | 3.6 | 0.021344 | 0.900396 | 36.15922 | 0.165593 |
| 1 | 2 : 1 | down | 4.2 | 0.0231 | 0.920483 | 34.72766 | 0.419422 |
| 1 | 2 : 1 | up | 4.2 | 0.011033 | 0.496711 | 3.947715 | 1 |
| 2 | 2 : 1 | down | 4.2 | 0.016391 | 0.81713 | 13.40512 | 1 |
| 2 | 2 : 1 | up | 4.2 | 0.015624 | 0.890487 | 32.5252 | 0.200939 |
| 1 | 1 : 1 | down | 4.8 | 0.022559 | 0.790602 | 11.32682 | 1 |
| 1 | 1 : 1 | up | 4.8 | 0.020276 | 0.741292 | 11.46146 | 1 |
| 2 | 1 : 1 | down | 4.8 | 0.026393 | 0.956277 | 65.6141 | 0.169076 |
| 2 | 1 : 1 | up | 4.8 | 0.020225 | 0.768589 | 13.28523 | 0.94041 |
| 1 | 1 : 2 | down | 5.4 | 0.128796 | 0.916524 | 32.93868 | 0.451672 |
| 1 | 1 : 2 | up | 5.4 | 0.082143 | 0.783997 | 14.51824 | 0.814248 |
| 2 | 1 : 2 | down | 5.4 | 0.08199 | 0.966717 | 87.13658 | 0.111929 |
| 2 | 1 : 2 | up | 5.4 | 0.078104 | 0.885253 | 30.85927 | 0.22102 |
| 1 | 2 : 1 | down | 6 | 0.063508 | 0.893026 | 25.04407 | 0.660265 |
| 1 | 2 : 1 | up | 6 | 0.045633 | 0.836463 | 20.45931 | 0.457176 |
| 2 | 2 : 1 | down | 6 | 0.089061 | 0.930983 | 40.46783 | 0.338023 |
| 2 | 2 : 1 | up | 6 | 0.046019 | 0.97196 | 138.6555 | **0.012814** |
| 1 | 1 : 1 | down | 6.7 | 0.13899 | 0.797272 | 11.79815 | 1 |
| 1 | 1 : 1 | up | 6.7 | 0.117654 | 0.831574 | 19.74925 | 0.485857 |
| 2 | 1 : 1 | down | 6.7 | 0.201368 | 0.917667 | 33.4374 | 0.442298 |
| 2 | 1 : 1 | up | 6.7 | 0.088852 | 0.865831 | 25.81315 | 0.304311 |
| 1 | 1 : 2 | down | 7.2 | -0.0076 | 0.020495 | 0.041847 | 1 |
| 1 | 1 : 2 | up | 7.2 | 0.112625 | 0.441135 | 1.578681 | 1 |
| 2 | 1 : 2 | up | 7.2 | -0.24198 | 0.495666 | 1.965622 | 1 |

**Supplementary Table 7:** Parameters of the intercept-only model (Equation 2), linear model (Equation 3), and broken-stick models (Equations 4 and 5) for the relationship between the competition coefficient and amplitude of temperature fluctuation (°C). Competition coefficients were calculated as the slopes of regression of the change in species abundance versus time (Equation 1) for pre-fluctuating (Pre), Fluctuating, and post-fluctuation (Post) stages of the experiment under both low and high N conditions. In the regression models *β_0_* is the intercept (i.e., the competition coefficient when temperature fluctuation amplitude (± °C) equals zero), *α_1_* is the breakpoint temperature fluctuation amplitude (± °C) at which the slope of the regression changes from *β_1_*  to *β_1_*+ *β_2_* and hence *β_2_* is the difference in slopes on either side of the threshold *α_1_*. The linear model does not have a break point. Where two breakpoints were identified, *α_2_* is the second breakpoint temperature fluctuation amplitude (± °C), at which the slope changes from *β_1_+β_2_ to β_1_+β_2_*+ *β_3_,* where *β_3_* is the difference in slopes either side of the second threshold *α_2._* In the case where no breakpoint was identified, or the broken stick model was less parsimonious linear regressions were used.

| **­­Stage** | | **N** | **Model** | **Equation** | ***β_0_*** | ***β_1_*** | ***β_2_*** | ***β_3_*** | **α_1_** | **α_2_** | **R^2^** | **Adj. R^2^** | **AIC** | **BIC** |
| --- | --- | --- | --- | --- | --- | --- | --- | --- | --- | --- | --- | --- | --- | --- |
| Pre | | Low | Intercept-only | 2 | 0.0014 ns |  |  |  |  |  | - | - | -485.1 | -480.7 |
|  | |  | Linear | 2 | -0.0020 ns | 0.0006* |  |  |  |  | 0.06 | 0.05 | -487.5 | -480.8 |
|  | |  | Broken stick | 3 | -0.0056 ns | 0.0015* | -0.0026* |  | 7.2 |  | 0.14 | 0.10 | -489.5 | -478.4 |
|  | | High | Intercept-only | 2 | -0.0015 * |  |  |  |  |  | - | - | -522.8 | -518.4 |
|  | |  | Linear | 2 | -0.0022 ns | 0.0001 ns |  |  |  |  | 0.00 | -0.01 | -521.1 | -514.5 |
|  | |  | Broken stick | n.a. | - | | | | | | 0.00 | -0.01 | -521.1 | -514.5 |
| Fluctuating | | Low | Intercept-only | 2 | -0.1535 *** |  |  |  |  |  | - | - | -32.62 | -28.18 |
|  | |  | Linear | 2 | 0.1686 *** | -0.0526*** |  |  |  |  | 0.73 | 0.72 | -118.7 | -112.0 |
|  | |  | Broken stick | 4 | 0.0197 ns | -0.0062 ns | -0.1441*** | 0.1539*** | 5.9 | 8.4 | 0.85 | 0.84 | -152.2 | -136.7 |
|  | | High | Intercept-only | 2 | -0.0717 *** |  |  |  |  |  | - | - | -96.59 | -92.15 |
|  | |  | Linear | 2 | 0.0113 *** | -0.03*** |  |  |  |  | 0.63 | 0.62 | -161.6 | -154.9 |
|  | |  | Broken stick | 3 | 0.0109 ns | -0.005*** | -0.1112*** |  | 8.2 |  | 0.96 | 0.96 | -304.9 | -293.8 |
| Post | | Low | Intercept-only | 2 | 0.0395 *** |  |  |  |  |  | - | - | -244.3 | -240.1 |
|  | |  | Linear | 2 | -0.0344* | 0.0180** |  |  |  |  | 0.49 | 0.47 | -162.1 | -156.9 |
|  | |  | Broken stick | n.a. | - | | | | | | 0.49 | 0.47 | -162.1 | -156.9 |
|  | | High | Intercept-only | 2 | 0.0148 *** |  |  |  |  |  | - | - | -131.2 | -127.8 |
|  | |  | Linear | 2 | -0.0156 ns | 0.0056*** |  |  |  |  | 0.23 | 0.21 | -257.6 | -251.3 |
|  | |  | Broken stick | n.a. | - | | | | | | 0.23 | 0.21 | -257.6 | -251.3 |
| \| ns indicates non significance at *p* ≥ 0.05. * indicates significance at *p* < 0.05, ** indicates significance at *p* < 0.01, *** indicates significance at *p* < 0.001, \| \| --- \| \| AIC is Akaike’s information criterion; BIC is the Schwarz Bayesian information criterion \| | | | | | | | | | | | | | | |
|  |  |  |  |  |  |  |  |  |  |  |  |  |  |  |

**Supplementary Table 8:** Estimates of the first and second breakpoints (α_1_ and α_2_, respectively) and approximate 95% confidence intervals (95%CI) for broken-stick models (with bootstrapping) across all experimental stages (Pre; pre-fluctuating, Fluctuating, and Post; post-fluctuation) under both low and high N conditions. Values in brackets are 95% CI.

| **Stage** | **N** | **α_1_** | **α_2_** |
| --- | --- | --- | --- |

| Pre | Low | 7.2 [4.8,9.6] | - |
| --- | --- | --- | --- |
|  | High | ns | - |
| Fluctuating | Low | 5.9 [5.3,6.5] | 8.4 [7.7,9.1] |
|  | High | 8.2 [8,8.5] |  |
| Post | Low | ns | - |
|  | High | ns | - |

**Supplementary Table 9:** Data of single slopes of *P.tricornutum*-to-*T.pseudonana* species abundance ratios over time used to calculate mean slopes over time in fluctuating environments in high N conditions and their confidence intervals (plotted in main text figure 3A). These mean slopes were used to calculate the number of days it would take to reach a 50% change in species abundance ratios with equation [3]. The time it would take is plotted in main text figure 4A.

| **Temperature Divergence from the Mean [°C]** | **Replicate 1 slope [day^-1^]** | **Replicate 2 slope [day^-1^]** | **Replicate 3 slope [day^-1^]** | **Replicate 4 slope [day^-1^]** | **Mean Slope [day^-1^]** | **Standard Deviation of Slope** | **Width of Confidence Interval (CI)** | **CI lower limit** | **CI upper limit** |
| --- | --- | --- | --- | --- | --- | --- | --- | --- | --- |
| 1.4 | -0.00097 | 0.004026 | 0.000437 | -0.00327 | 5.58E-05 | 0.003056 | 0.002995 | -0.00294 | 0.003051 |
| 1.9 | -0.00168 | 0.013813 | 0.000923 | -0.01298 | 1.9E-05 | 0.010999 | 0.010779 | -0.01076 | 0.010798 |
| 2.4 | -0.00843 | 0.000324 | 0.002583 | -0.00095 | -0.00162 | 0.00477 | 0.004675 | -0.00629 | 0.003057 |
| 3.1 | -0.00507 | -0.00183 | -0.00945 | -0.00881 | -0.00629 | 0.003546 | 0.003475 | -0.00976 | -0.00282 |
| 3.5 | -0.03245 | 0.014117 | -0.00461 | -0.00903 | -0.00799 | 0.019144 | 0.018761 | -0.02675 | 0.010768 |
| 4.2 | -0.00532 | 0.002341 | -0.00197 | -0.01432 | -0.00482 | 0.007069 | 0.006927 | -0.01174 | 0.00211 |
| 4.7 | -0.01472 | -0.0032 | -0.01367 | -0.01956 | -0.01279 | 0.006887 | 0.006749 | -0.01954 | -0.00604 |
| 5.4 | -0.01542 | 0.000666 | -0.00699 | -0.01748 | -0.00981 | 0.008327 | 0.00816 | -0.01797 | -0.00165 |
| 5.9 | -0.01131 | -0.00124 | -0.00892 | -0.02314 | -0.01115 | 0.009073 | 0.008892 | -0.02004 | -0.00226 |
| 6.4 | -0.01765 | -0.01448 | -0.01606 | -0.01842 | -0.01665 | 0.00175 | 0.001715 | -0.01837 | -0.01494 |
| 7.2 | -0.06065 | -0.01837 | -0.04853 | -0.03158 | -0.03978 | 0.018599 | 0.018227 | -0.05801 | -0.02156 |
| 7.8 | -0.02579 | -0.02223 | -0.01796 | -0.05367 | -0.02991 | 0.016159 | 0.015835 | -0.04575 | -0.01408 |
| 8.5 | -0.06398 | -0.05116 | -0.04449 | -0.10837 | -0.067 | 0.028741 | 0.028166 | -0.09517 | -0.03883 |
| 9.1 | -0.17695 | -0.1382 | -0.15406 | -0.19552 | -0.16618 | 0.02521 | 0.024705 | -0.19089 | -0.14148 |
| 9.7 | -0.17383 | -0.09608 | -0.17471 | -0.20408 | -0.16218 | 0.046251 | 0.045325 | -0.2075 | -0.11685 |
| 10.4 | -0.22104 | -0.29297 | -0.27592 | -0.24031 | -0.25756 | 0.032773 | 0.032117 | -0.28968 | -0.22544 |
| 11.4 | -0.41616 | -0.4683 | -0.39704 | -0.41986 | -0.42534 | 0.030336 | 0.029728 | -0.45507 | -0.39561 |

**Supplementary Table 10:** Data of single slopes of *P.tricornutum*-to-*T.pseudonana* species abundance ratios over time used to calculate mean slopes over time in fluctuating environments in low N conditions and their confidence intervals (plotted in main text figure 3B). These mean slopes were used to calculate the number of days it would take to reach a 50% change in species abundance ratios with equation [3]. The time it would take is plotted in main text figure 4A.

| **Temperature Divergence from the Mean [°C]** | **Replicate 1 slope [day^-1^]** | **Replicate 2 slope [day^-1^]** | **Replicate 3 slope [day^-1^]** | **Replicate 4 slope [day^-1^]** | **Mean Slope [day^-1^]** | **Standard Deviation of Slope** | **Width of Confidence Interval (CI)** | **CI lower limit** | **CI upper limit** |
| --- | --- | --- | --- | --- | --- | --- | --- | --- | --- |
| 1.4 | 0.006803 | 0.007418 | 0.005363 | 0.004811 | 0.006099 | 0.001216 | 0.001192 | 0.004907 | 0.00729 |
| 2 | 0.00925 | -0.00287 | 0.015589 | -0.00151 | 0.005115 | 0.00884 | 0.008663 | -0.00355 | 0.013778 |
| 2.6 | 0.004704 | 0.00571 | 0.003258 | 0.003061 | 0.004183 | 0.001254 | 0.001229 | 0.002954 | 0.005412 |
| 3.1 | 0.008249 | 0.011138 | 0.0071 | 0.007253 | 0.008435 | 0.001873 | 0.001835 | 0.0066 | 0.01027 |
| 3.6 | 0.004377 | 0.002062 | 0.005344 | -0.00106 | 0.002681 | 0.002849 | 0.002792 | -0.00011 | 0.005472 |
| 4.2 | -0.00169 | -0.00046 | -0.00481 | -0.00367 | -0.00266 | 0.001951 | 0.001912 | -0.00457 | -0.00075 |
| 4.8 | -0.00813 | -0.00796 | -0.01082 | -0.01319 | -0.01003 | 0.002484 | 0.002434 | -0.01246 | -0.00759 |
| 5.4 | -0.03746 | -0.03351 | -0.03835 | -0.03526 | -0.03615 | 0.002185 | 0.002141 | -0.03829 | -0.034 |
| 6 | -0.04552 | -0.04123 | -0.04591 | -0.04191 | -0.04364 | 0.002414 | 0.002366 | -0.04601 | -0.04128 |
| 6.7 | -0.10954 | -0.08846 | -0.14062 | -0.10638 | -0.11125 | 0.021669 | 0.021235 | -0.13249 | -0.09001 |
| 7.2 | -0.20236 | -0.20316 | -0.2968 | -0.18808 | -0.2226 | 0.049949 | 0.04895 | -0.27155 | -0.17365 |
| 7.8 | -0.18385 | -0.25359 | -0.2557 | -0.24411 | -0.23431 | 0.034017 | 0.033336 | -0.26765 | -0.20098 |
| 8.4 | -0.48317 | -0.5693 | -0.49645 | -0.4905 | -0.50986 | 0.04 | 0.0392 | -0.54905 | -0.47066 |
| 9.1 | -0.29692 | -0.34638 | -0.30742 | -0.36988 | -0.33015 | 0.033974 | 0.033294 | -0.36344 | -0.29686 |
| 9.8 | -0.23024 | -0.30603 | -0.23871 | -0.3179 | -0.27322 | 0.045133 | 0.04423 | -0.31745 | -0.22899 |
| 10.5 | -0.36904 | -0.67626 | -0.31617 | -0.63056 | -0.49801 | 0.181697 | 0.17806 | -0.67607 | -0.31995 |
| 11.4 | -0.27432 | -0.45734 | -0.31538 | -0.41034 | -0.36435 | 0.084194 | 0.082508 | -0.44685 | -0.28184 |

**Supplementary Table 11:** Data of single slopes of *P.tricornutum*-to-*T.pseudonana* species abundance ratios over time used to calculate mean slopes over time in stable temperature environments in high N conditions (Siegel et al. 2020). These mean slopes were used to calculate the number of days it would take to reach a 50% change in species abundance ratios with equation [3]. The time it would take is plotted in main text figure 4B.

| **Assay temperature in stable environment [°C]** | **Replicate 1 slope [day^-1^]** | **Replicate 2 slope [day^-1^]** | **Replicate 3 slope [day^-1^]** | **Replicate 4 slope [day^-1^]** | **Mean Slope [day^-1^]** | **Standard Deviation of Slope** |
| --- | --- | --- | --- | --- | --- | --- |
| 8.8 | 0.220016 | 0.180301 | 0.242393 | 0.227091 | 0.21745 | 0.026469 |
| 10.7 | 0.193831 | 0.17363 | 0.142858 | 0.197262 | 0.176895 | 0.024972 |
| 12.3 | 0.143597 | 0.196603 | 0.144162 | 0.12191 | 0.151568 | 0.03176 |
| 14 | 0.125312 | 0.128951 | 0.11085 | 0.130095 | 0.123802 | 0.008872 |
| 15.6 | 0.075963 | 0.075415 | 0.092893 | 0.079143 | 0.080854 | 0.008193 |
| 17.3 | 0.026093 | -0.00376 | 0.082083 | 0.055003 | 0.039855 | 0.036988 |
| 19 | -0.01354 | -0.05187 | 0.097808 | 0.005794 | 0.009547 | 0.063534 |
| 20.5 | -0.104 | -0.06126 | 0.001121 | -0.08948 | -0.06341 | 0.046535 |
| 22.2 | -0.12047 | -0.12394 | -0.13411 | -0.06762 | -0.11153 | 0.029845 |
| 23.6 | -0.13515 | -0.18098 | -0.08798 | -0.17298 | -0.14427 | 0.042521 |
| 25.3 | -0.17421 | -0.23516 | -0.2055 | -0.35523 | -0.24252 | 0.079148 |
| 26.8 | -0.33769 | -0.3631 | -0.40278 | -0.44345 | -0.38675 | 0.046323 |
| 28.5 | -0.58652 | -0.76936 | -0.60297 | -0.57676 | -0.6339 | 0.090948 |
| 29.9 | -0.73022 | -0.89579 | NA | NA | -0.81301 | 0.117076 |
| 31.5 | -0.96312 | -0.73406 | NA | NA | -0.84859 | 0.161974 |
| 33.1 | -0.93633 | -0.73192 | NA | NA | -0.83413 | 0.144539 |
| 35 | -0.71206 | -0.88202 | NA | NA | -0.79704 | 0.120177 |

**Supplementary Table 12:** Data of single slopes of *P.tricornutum*-to-*T.pseudonana* species abundance ratios over time used to calculate mean slopes over time in stable temperature environments in low N conditions (Siegel et al. 2020). These mean slopes were used to calculate the number of days it would take to reach a 50% change in species abundance ratios with equation [3]. The time it would take is plotted in main text figure 4B.

| **Assay temperature in stable environment [°C]** | **Replicate 1 slope [day^-1^]** | **Replicate 2 slope [day^-1^]** | **Replicate 3 slope [day^-1^]** | **Replicate 4 slope [day^-1^]** | **Mean Slope [day^-1^]** | **Standard Deviation of Slope** |
| --- | --- | --- | --- | --- | --- | --- |
| 9.1 | 0.264448 | 0.285903 | NA | NA | 0.275176 | 0.015171 |
| 11.1 | 0.169038 | 0.157061 | 0.242232 | 0.255092 | 0.205856 | 0.049946 |
| 12.9 | 0.078705 | 0.112903 | 0.167966 | 0.161986 | 0.13039 | 0.042377 |
| 14.6 | 0.106816 | 0.095109 | 0.106283 | 0.144045 | 0.113063 | 0.021348 |
| 16 | 0.074248 | 0.061848 | 0.030937 | 0.047922 | 0.053739 | 0.018621 |
| 17.4 | 0.034376 | 0.054908 | 0.068885 | 0.064853 | 0.055755 | 0.015416 |
| 19.1 | 0.051233 | 0.044168 | 0.075651 | 0.058107 | 0.05729 | 0.013499 |
| 20.5 | 0.033114 | 0.04143 | 0.090213 | 0.092059 | 0.064204 | 0.031292 |
| 22 | 0.050768 | 0.025887 | 0.114819 | 0.085633 | 0.069276 | 0.039017 |
| 23.6 | 0.043563 | 0.041392 | 0.046228 | 0.005526 | 0.034177 | 0.019203 |
| 25 | 0.008992 | 0.016378 | 0.011949 | 0.120911 | 0.039557 | 0.05432 |
| 26.5 | -0.08148 | -0.05256 | 0.038533 | 0.076785 | -0.00468 | 0.074598 |
| 28 | -0.24175 | -0.19793 | -0.11477 | -0.36877 | -0.23081 | 0.105983 |
| 29.4 | -0.35949 | NA | NA | NA | -0.35949 | NA |

## Supplementary Figures


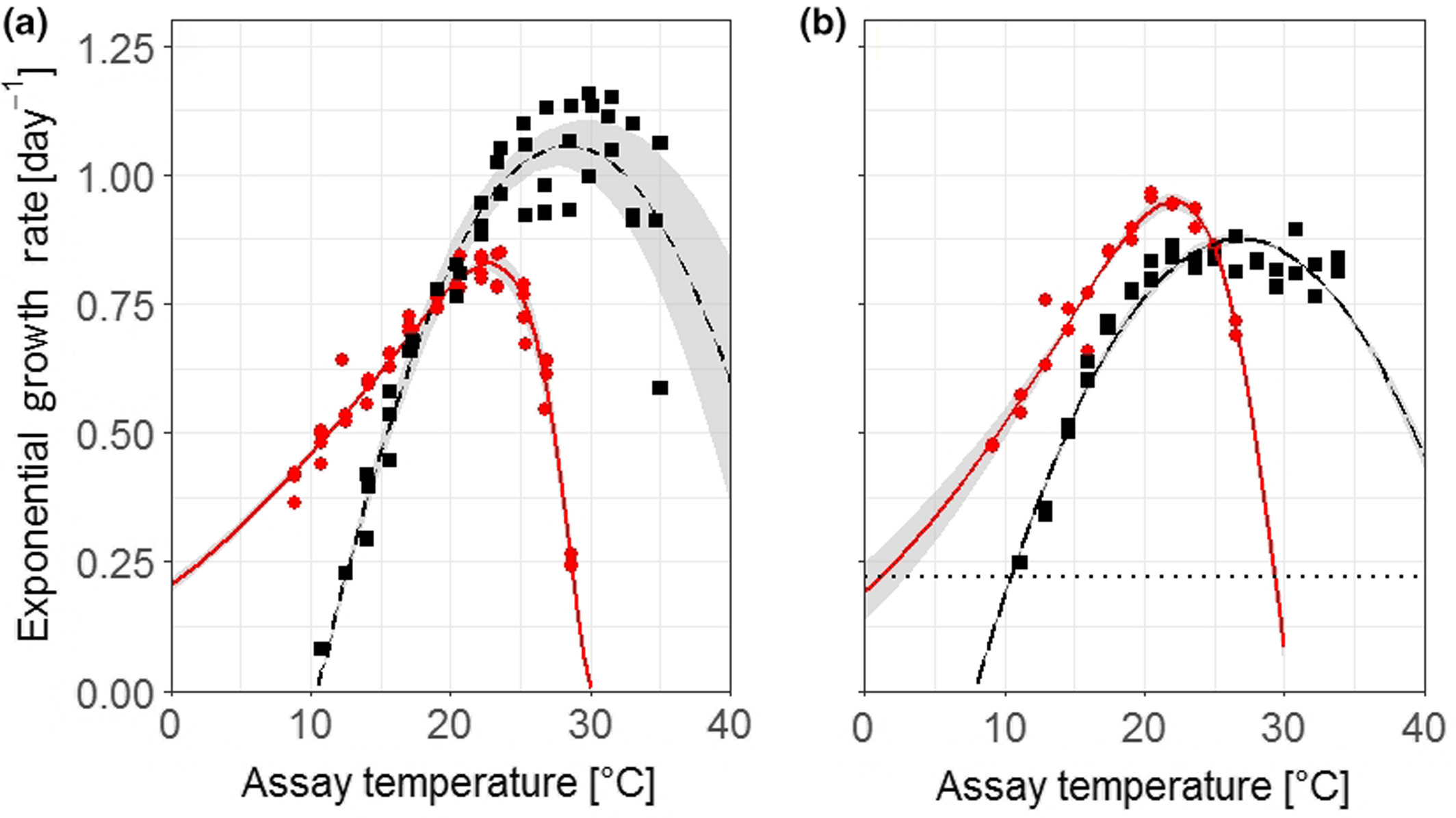


**Supplementary Figure 1**: Reproduced from Figure 2 from Siegel et al. (2020). Average temperature response curves depicting (a) acclimated growth rates of *Phaeodactylum tricornutum* (red circles and solid line) and *Thalassiosira pseudonana* (black squares and dashed line) under high nitrate conditions (*n* = 4 and 3 respectively) and (b) the initial acute growth rate (days 0–4) under low N conditions (*n* = 2 for both species). Shaded area denotes standard deviation of the average model calculated from replicate model fits. Symbols in (a) represent the growth rates from distinct biological replicates and were calculated from the average growth rates across dilution steps after the diatoms had acclimated to growth under assay conditions. Dotted line in (b) indicates ln(1/0.8), the daily dilution rate that was imposed by the sampling regime in these semicontinuous cultures; cell abundance would have declined in cultures with growth rates below this value which equates to a growth rate of 0.22 d^−1^.


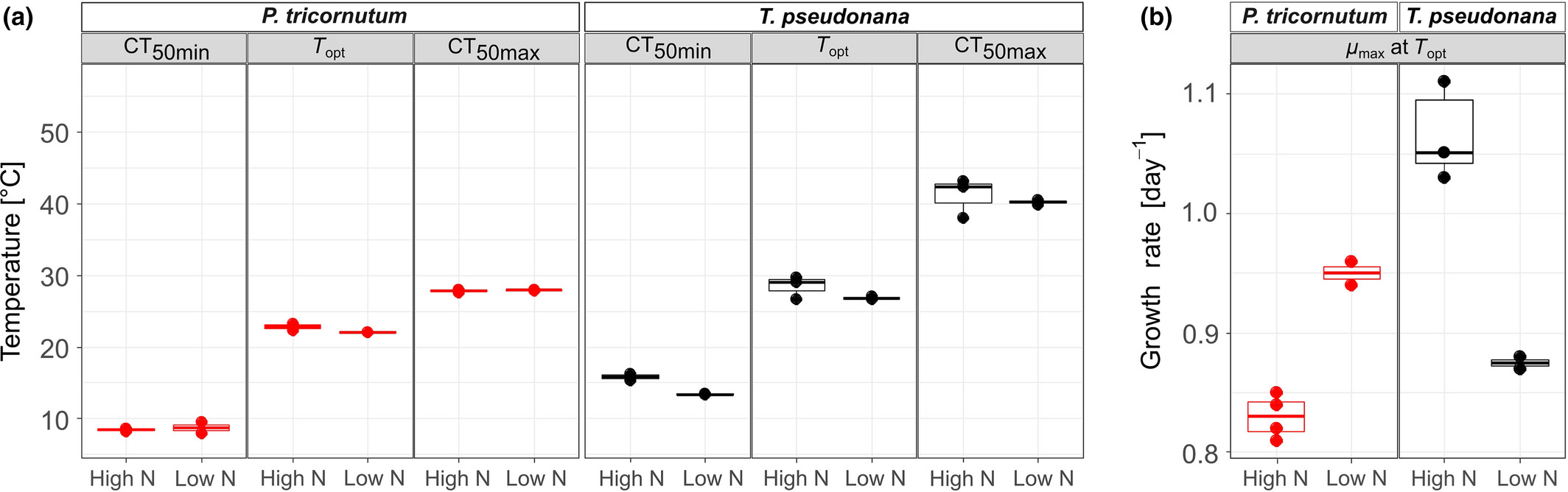


**Supplementary Figure 2**: Reproduced from Figure 3 from Siegel et al. (2020). Comparison of (a) cardinal temperatures and (b) maximum growth rates from thermal performance curve model fits for growth rates between *Phaeodactylum tricornutum* and *Thalassiosira pseudonana* under high N and low N growth conditions. For *Phaeodactylum tricornutum,* *n* = 4 in high N and *n* = 2 in low N. For *Thalassiosira pseudonana,* *n* = 3 in high N and *n* = 2 in low N.


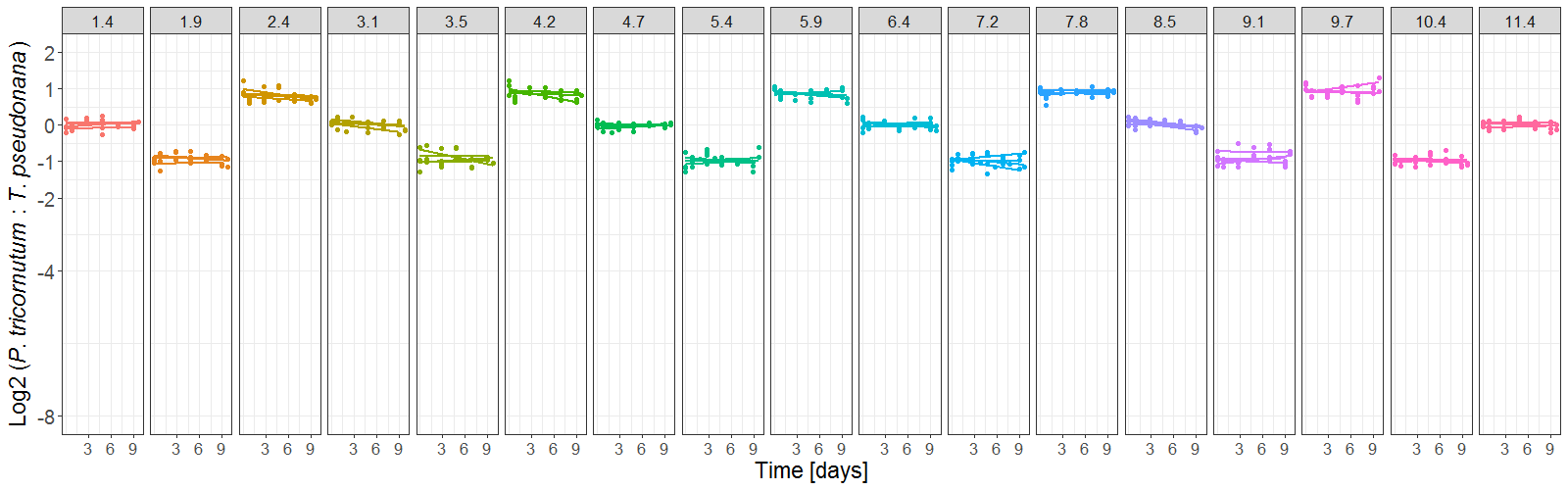


Supplementary Figure 3: Progression of log2(*P.tricornutum* : *T. pseudonana*) over time in high N mixed cultures at 18.6 °C before temperature fluctuations were started. Each panel displays 4 replicate cultures and the temperature divergence from the temperature of coexistence they were exposed to after this 10 day pre-fluctuation period. Log_2_ was utilised in this figure, and in the following 5 supplementary figures, as a biologically relevant depiction of species frequency change over time, whereby a doubling or halving in the abundance ratio translates to one unit on the log_2_ scale.


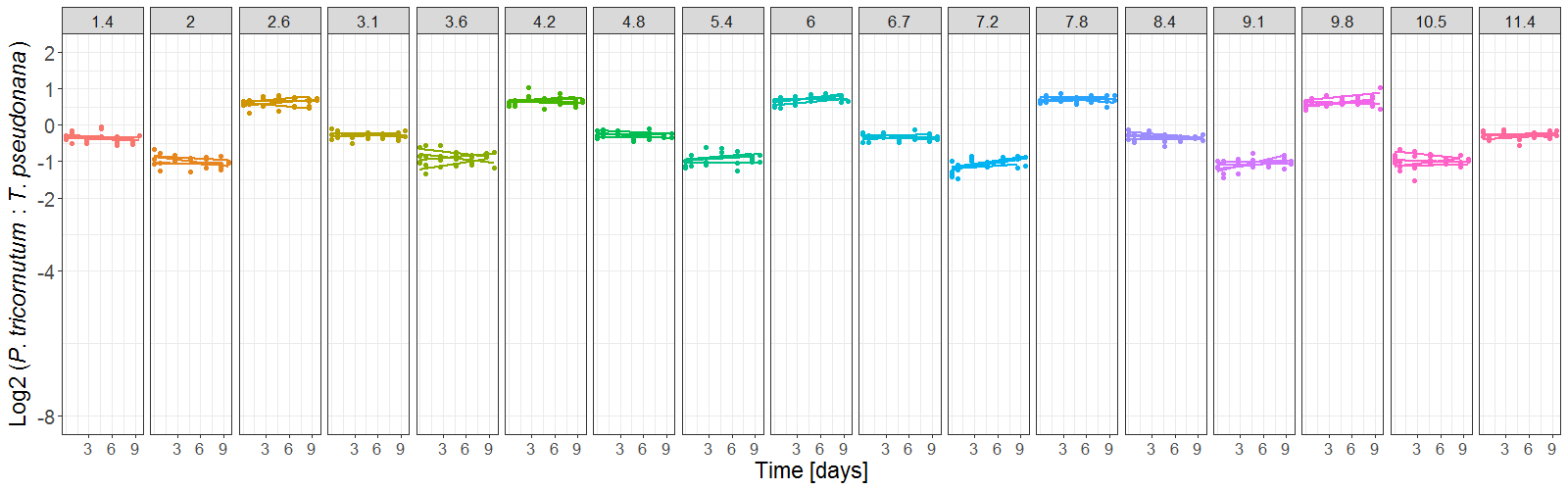


Supplementary Figure 4: Progression of log2(*P.tricornutum* : *T. pseudonana*) over time in low N mixed cultures at 25.2 °C before temperature fluctuations were started. Each panel displays 4 replicate cultures and the temperature divergence from the temperature of coexistence they were exposed to after this 10 day pre-fluctuation period.


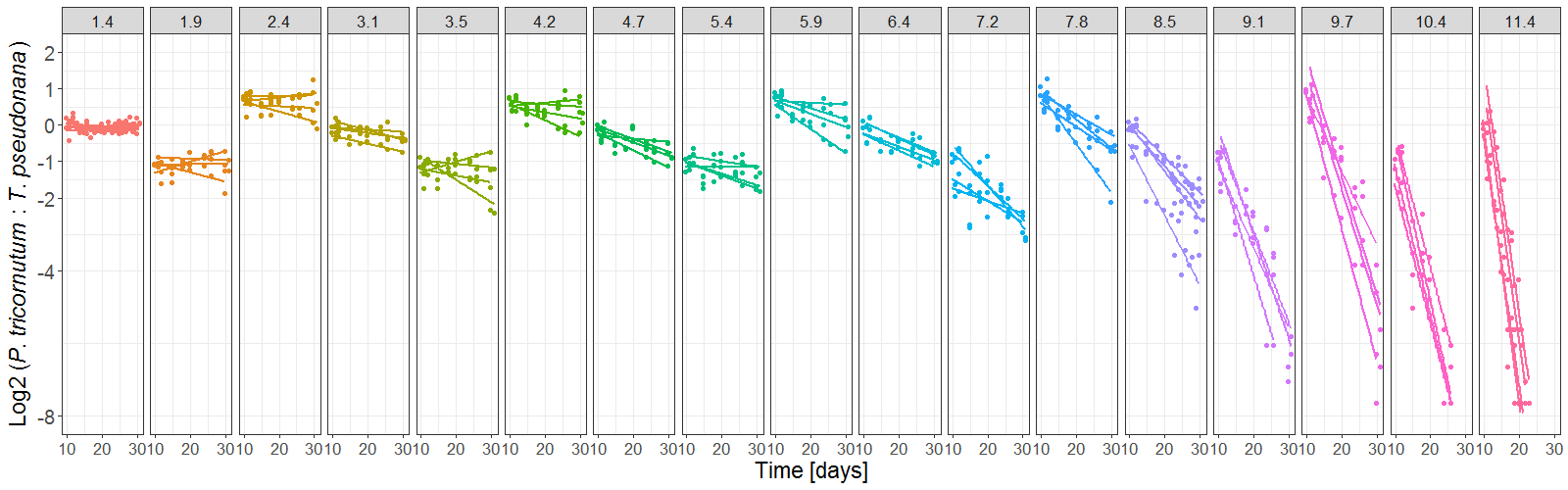


Supplementary Figure 5: Progression of log2(*P.tricornutum* : *T. pseudonana*) species ratio over time in mixed cultures at different fluctuation amplitudes during the fluctuation period of the high N experiment. Each panel represents an assay temperature divergence around the mean temperature of 18.6 °C.


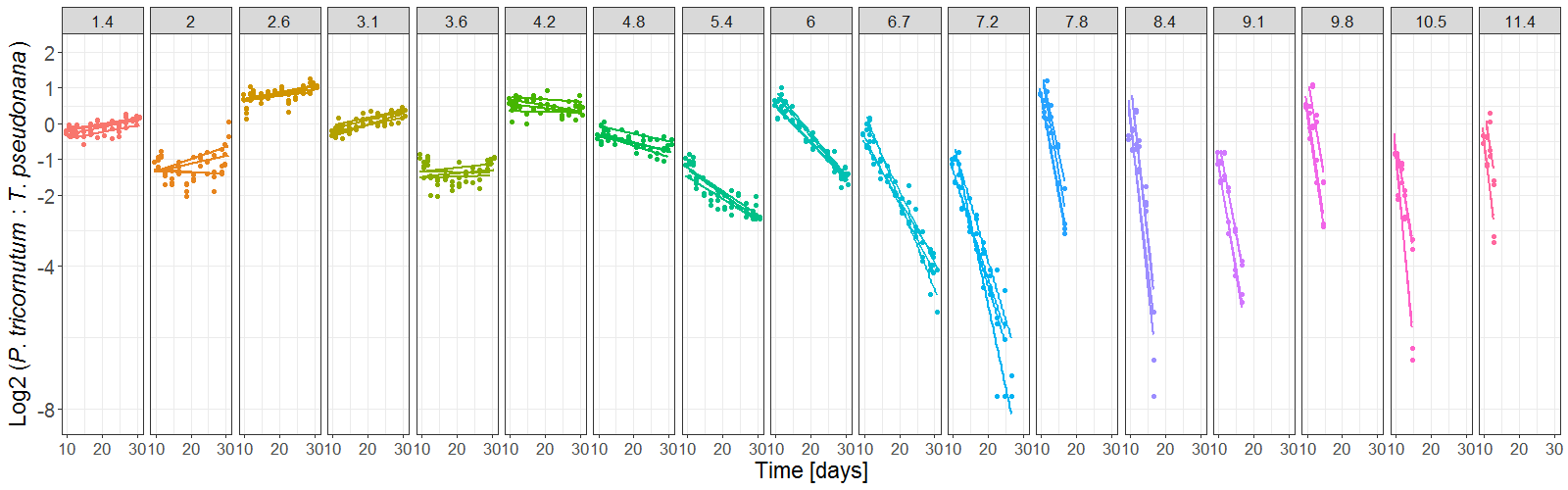


Supplementary Figure 6: Progression of log2(*P.tricornutum* : *T. pseudonana*) species ratio over time in mixed cultures at different fluctuation amplitudes during the fluctuation period of the low N experiment. Each panel represents an assay temperature divergence around the mean temperature of 25.2 °C.


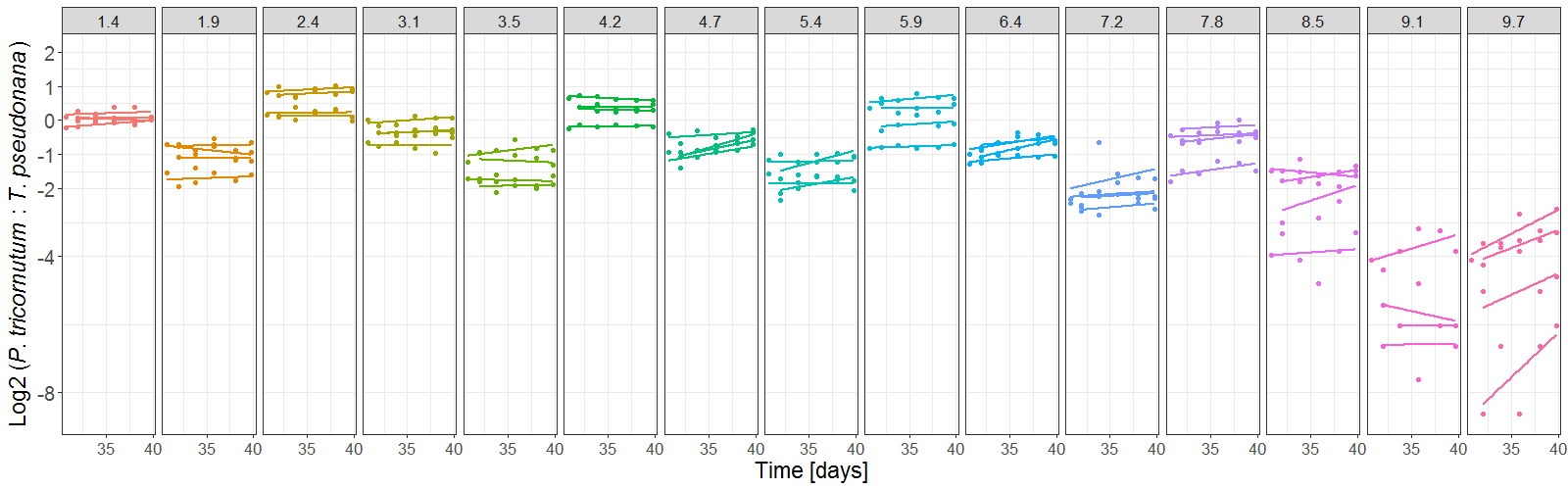


Supplementary Figure 7: Progression of log2(*P.tricornutum* : *T. pseudonana*) species ratio over time in mixed cultures at different fluctuation amplitudes after the fluctuation period of the high N experiment. Each panel represents an assay temperature divergence around the mean temperature of 18.6 °C that the mixed cultures were exposed to during the temperature fluctuation period.


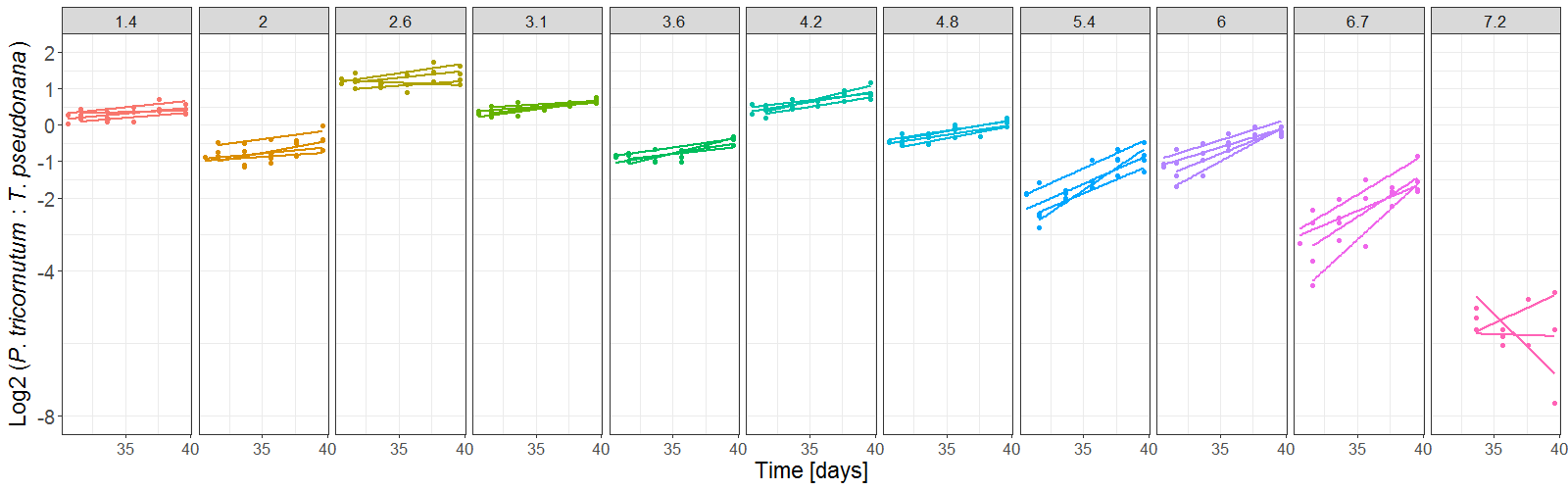


Supplementary Figure 8: Progression of log2(*P.tricornutum* : *T. pseudonana*) species ratio over time in mixed cultures at different fluctuation amplitudes after the fluctuation period of the low N experiment. Each panel represents an assay temperature divergence around the mean temperature of 25.2 °C that the mixed cultures were exposed to during the temperature fluctuation period.


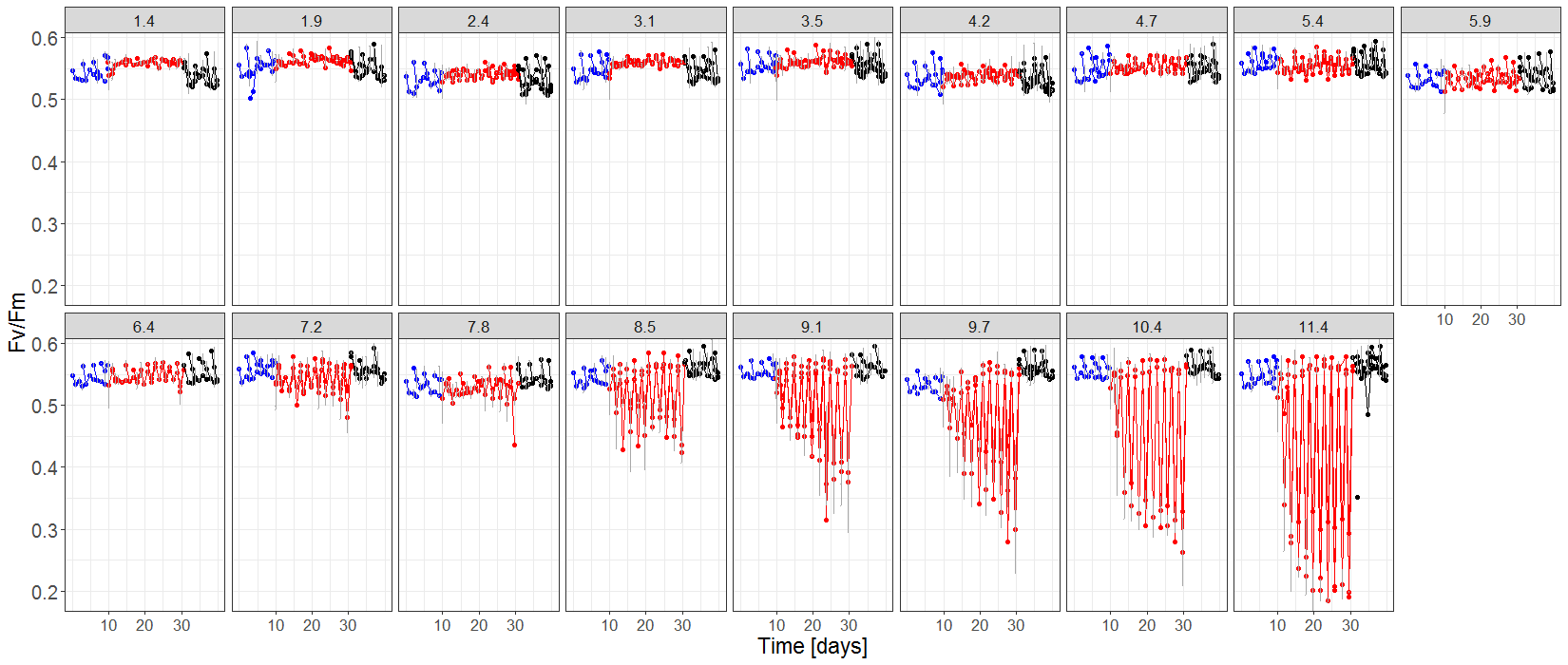


Supplementary Figure 9: Average (n = 4) *F_v_*/*F_m_* of exponentially growing mixed cultures of *P. tricornutum* and *T. pseudonana* across temperature fluctuation amplitudes. Blue data indicate the period prior to the start of the fluctuations, red data indicate the fluctuation period, and black data indicate the period after fluctuations. Each panel represents one assay fluctuation amplitude around the mean temperature of 18.6 °C. Note how at the highest fluctuation temperatures (17 °C and above), a general drop in values in comparison to the pre-fluctuation period (blue) can be observed in the first three to five days of the fluctuation period (red). During this time, *P. tricornutum* encountered conditions close to its physiological limits on the warm cycles, and *T. pseudonana* on the cold cycles. As the fluctuation period progressed and P. *tricornutum* was fully or almost fully displaced from the mixed cultures at high fluctuation amplitudes, this drop in values could only be observed every second day of the experiment. This is because *T. pseudonana* encountered unfavourable conditions on cold days, but not on warm days, and *P. tricornutum* did not influence the *F_v_*/*F_m_* signal anymore.

**Limitations of the study**

As the species could not be separated from each other during the time they were cultured together, it was impossible to assess the health of single species throughout the experiment. Regardless of this shortcoming, it is however possible to make certain inferences based on their physiological performance as monocultures, which were investigated in Siegel et al. (2020). Temperatures above 30 °C where lethal for *P. tricornutum*, consistent with the observation that *F_v_*/*F_m_* declined to 0 over time in monocultures exposed to a range of 30 °C to 33 °C. Fluctuations to 33 °C or beyond would have killed *P. tricornutum* within one 24-hour-cycle, as *F_v_*/*F_m_* in monocultures dropped to 0 within one day in *P. tricornutum* monocultures at these temperatures. It can therefore be said with certainty that *P. tricornutum* cells in assay cultures reaching more than 33 °C were dead after one warm fluctuation cycle. Live and dead cells were not distinguishable in the manual Lugol’s counts and small numbers of *P. tricornutum* cells could have influenced the calculated species ratios strongly. This likely accounts for the large scatter in the competition coefficients that were observed for fluctuation amplitudes that reached the lethal temperature range for *P. tricornutum* on warm days (grey-shaded are in Fig. 3B). Likewise to *P. tricornutum* reaching its limits at the warm extremes, cold temperatures below 10 °C were stressful to *T. pseudonana*, which must have been struggling to survive at this and lower temperatures. Further evidence of this can be seen in Supplementary Figure 7, where *F_v_*/*F_m_* in high N cultures dropped severely on every cold fluctuation cycle at fluctuation amplitudes of 17 °C or higher.

Towards the end of the low N experiment, particularly during the period after temperature fluctuations, empty frustules were sometimes observed and *T. pseudonana* cells began to aggregate in clumps the longer the experiment continued. This could be attributed to a general decline in cell health due to being cultured under nutrient limitation for 40 days (almost 6 weeks). The significant trends in species frequencies during the post-fluctuation period in favour of *P. tricornutum* could be partially attributed to clumping of *T. pseudonana* cells (Main text Fig. 3C, Suppl. Fig. 8). Single cells of *T. pseudonana* became less distinguishable and cell counts less accurate, underestimating the number of *T. pseudonana* cells present in the cultures. Therefore, it is possible that more cultures would have been stable again after fluctuations stopped, as in the case of the high N scenario, if cultures had not been struggling under the pressure of continuous N-depletion.
